# Trap Configuration and Spacing Influences Parameter Estimates in Spatial Capture-Recapture Models

**DOI:** 10.1371/journal.pone.0088025

**Published:** 2014-02-05

**Authors:** Catherine C. Sun, Angela K. Fuller, J. Andrew Royle

**Affiliations:** 1 New York Cooperative Fish and Wildlife Research Unit, Department of Natural Resources, Cornell University, Ithaca New York, United States of America; 2 U.S. Geological Survey, New York Cooperative Fish and Wildlife Research Unit, Department of Natural Resources, Cornell University, Ithaca, New York, United States of America; 3 U.S. Geological Survey, Patuxent Wildlife Research Center, Laurel, Maryland, United States of America; University of Kent, United Kingdom

## Abstract

An increasing number of studies employ spatial capture-recapture models to estimate population size, but there has been limited research on how different spatial sampling designs and trap configurations influence parameter estimators. Spatial capture-recapture models provide an advantage over non-spatial models by explicitly accounting for heterogeneous detection probabilities among individuals that arise due to the spatial organization of individuals relative to sampling devices. We simulated black bear (*Ursus americanus)* populations and spatial capture-recapture data to evaluate the influence of trap configuration and trap spacing on estimates of population size and a spatial scale parameter, sigma, that relates to home range size. We varied detection probability and home range size, and considered three trap configurations common to large-mammal mark-recapture studies: regular spacing, clustered, and a temporal sequence of different cluster configurations (i.e., trap relocation). We explored trap spacing and number of traps per cluster by varying the number of traps. The clustered arrangement performed well when detection rates were low, and provides for easier field implementation than the sequential trap arrangement. However, performance differences between trap configurations diminished as home range size increased. Our simulations suggest it is important to consider trap spacing relative to home range sizes, with traps ideally spaced no more than twice the spatial scale parameter. While spatial capture-recapture models can accommodate different sampling designs and still estimate parameters with accuracy and precision, our simulations demonstrate that aspects of sampling design, namely trap configuration and spacing, must consider study area size, ranges of individual movement, and home range sizes in the study population.

## Introduction

Estimating population parameters such as abundance and density is crucial for understanding, managing, and conserving animal populations. Capture-mark-recapture (CMR) methods are a well-established approach in which repeated sampling with replacement of a population provides information about detection probabilities of individuals. CMR models have become increasingly realistic by addressing assumptions about population closure and capture probability [1]–[4], including the recent developments of spatial capture-recapture (SCR) models. SCR models incorporate the geographic locations where individuals are detected, thereby explicitly accounting for unequal detection probabilities among individuals due to their unique spatial locations relative to sampling devices (traps, snares, etc.) [5], [6]. Unequal exposure of individuals to the sampling array occurs when, for example, some individuals have home ranges at the edge of the sampling array while others are located more centrally and therefore are always exposed to the sampling array [2], [4], [7], [8]. As a result, non-spatial capture-recapture methods estimate population size, but require various ad-hoc approaches to convert estimates of population size to estimates of density. Non-spatial approaches attempt to homogenize the unequal trap exposure with methods such as minimizing the ratio of edge to area of the sampling grid [9], or by adding a buffer strip around the sampling array to account for movements of ‘edge’ individuals [10]–[13]. Conversely, SCR models directly estimate both population size and density; SCR models allow for individual-specific detection probabilities by accounting for the spatial organization of traps and by estimating the activity centers of individuals. SCR models are thus liberated from the assumption of geographic closure.

Two primary considerations of mark-recapture sampling design are the spatial extent of the trap array and the spacing between traps. An advantage of a large spatial extent is that it helps increase the expected number of unique individuals detected. For non-spatial approaches, Bondrup-Nielson [9] suggested that the spatial extent of a study area be at least four times the home range size of an individual. Large spatial extents also aim to capture the full range of movement of individuals and homogenize unequal detection rates among individuals [9], [14], [15]. Simultaneously, trap spacing influences rates of detection and recaptures: trap configurations with “holes”, or traps that are too widely spaced relative to ranges of individual movement, can lead to individuals not being detected [12], [16] as well as fewer recaptures of individuals at different traps (i.e., spatial recaptures), which are important for estimating home range sizes and movement ranges [15]. As a result, recommendations have been made to set at least four traps in each potential non-overlapping home range [17]. With a constant number of traps due to logistical or monetary considerations, a sampling trade-off occurs between spatial extent and trap spacing.

Few simulation-based studies have been conducted on the influence of sampling design on SCR parameter estimates [15], [18], [19]. SCR approaches can theoretically accommodate different spatial arrangements of traps because trap locations are a formal part of the model [5] describing the probability of encounter of individuals. However, spatial organization of the trapping array is still an important consideration. Large numbers of encountered individuals (sample size *n)* and recaptures are necessary to estimate population parameters with accuracy and precision, and trap arrangements need to be at spatial densities and scales that permit detection of individual movement [15]. Much of the early research on sampling design was conducted in small mammal community assemblages [20]–[22], and design recommendations based on small mammal populations may be hard to meet and are sometimes inappropriate for large-mammal systems [12], [19]. Recent work has focused specifically on sampling designs for large-mammal populations with large home ranges and ranges of movement [15], [16], [23], [24], but the body of published research remains scant. Notably, Sollmann et al. [15] demonstrated with simulations and a study of a Michigan black bear population that previously recommended spatial extents of at least 4× the home range size of individuals may be unnecessary. The authors showed that spatial extents smaller than an average male home range and only 1.5× larger than a female’s yielded parameter estimates similar to when the full spatial extent was used. The authors cautioned that the range of movement over the sampling array is important for SCR models, and that the SCR model performed well as long as the spatial scale parameter sigma (σ) was at least half the average trap spacing. This sigma parameter describes the spatial scale over which an individual is detected, and can be converted to an estimate of the 95% home range radius [14]. The authors concluded that SCR models are able to accurately and precisely estimate population parameters for a range of sampling array extents, but that more research is necessary to explore the limits of SCR abilities with respect to trap configuration and extreme sampling designs.

Understanding the implications of different sampling designs is crucial, especially given the amount of effort required in large mammal mark-recapture studies and the increasing application of SCR methods. Moreover, in sampling over large landscapes, it is oftentimes not possible to achieve regular coverage of the landscape with traps that are close enough together to yield sufficient data for effective parameter estimation. Therefore, strategies for distributing traps over the landscape in an efficient manner must be developed and evaluated. To improve understanding of sampling design with respect to SCR methods, we conducted simulations to investigate the effects of different trap configurations and spacings that would be feasible in large-mammal studies. First, we evaluated potential differences among three common trap configurations: regular spacing, clustered, and a temporal sequence of different clustered configurations (i.e., trap relocation) [25]. The regular trap configuration, in which traps are set systematically across the spatial extent, served as a baseline for comparison. The clustered configuration maintained spatially representative sampling over the entire spatial extent while providing more information on the spatial scale of detection and individual movement [17], [19]. The third configuration evaluated was a clustered configuration with trap relocation midway through sampling; trap relocation is a common sampling approach to increase detection probability, more thoroughly sample large study areas, and avoid trap habituation and behavioral response [26]. Our second objective was to identify consequences of trap spacing for a fixed study area by decreasing the number of traps while maintaining the same spatial extent of the sampling array. We conducted our simulation study using sampling design considerations for American black bear (*Ursus americanus*), but the results are generalizable to any wide-ranging animal population to which SCR models might be applicable.

## Methods

**Figure 1:**
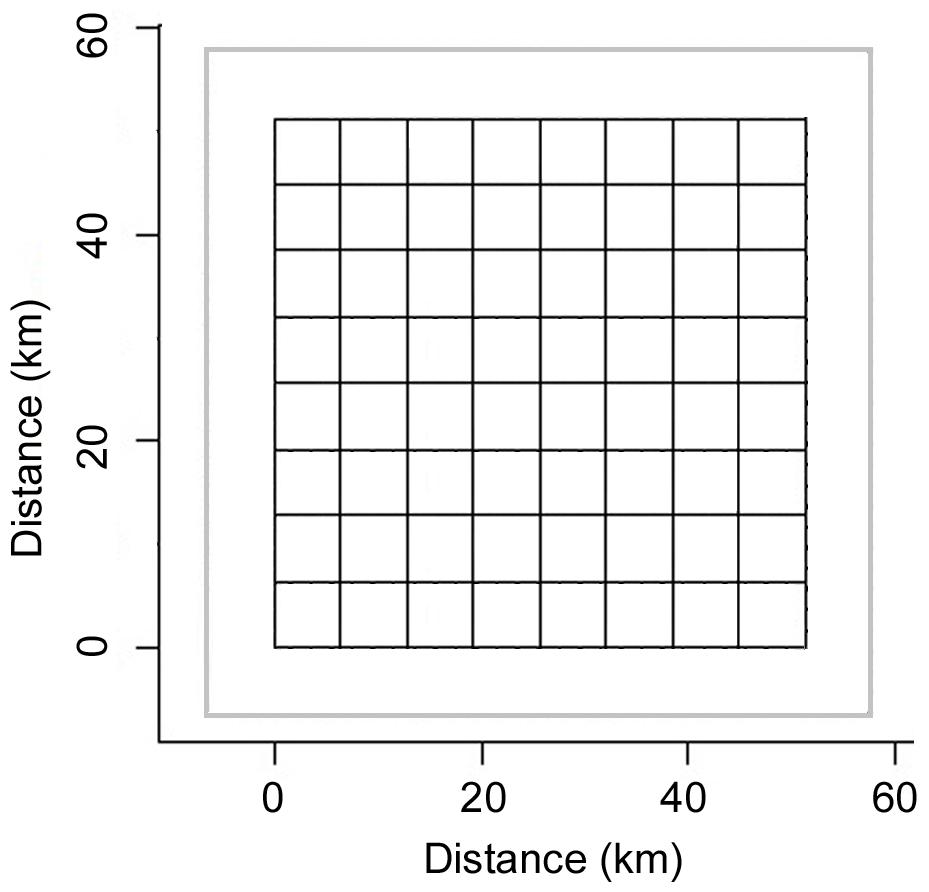
Schematic representation of study area. A representation of the 2,264 km^2^ study area, divided into a grid of 64 cells of 41 km^2^ each, and set in the center of a 4,100 km^2^ landscape, which is outlined in gray.

We based simulation conditions on characteristics of a black bear population study conducted in southwestern New York, USA. The simulated study area was a 2,624 km^2^ square centered on a 4,100 km^2^ landscape. To determine trap placement in the clustered and sequential trap configurations, we overlaid a grid of 64, non-overlapping, potential home ranges of 41 km^2^ each, based on the average female home range size estimated in northwestern Pennsylvania [27] (Figure 1).

### SCR Model Formulation and Implementation

We used a binomial model for detection to create encounter histories for individuals. For sampling over *K* sampling periods, the number of encounters for an individual, *i,* in each of *j = 1,…, J* traps, *y_i_*_j_, has a binomial distribution with a parameter for encounter probability, *p_ij_*. In other words:
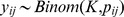



For the individual and trap-specific encounter probability, *p_ij_*, we used the half-normal model [28], which depends on the baseline detection probability
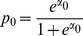
and a function of the Euclidean distance, *D_ij_*, between individual, *i,* and trap, *j*, such that



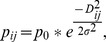
where *σ* is a spatial scale parameter determining the rate of decrease in encounter probability as a function of distance to trap *D_ij_.* Most models for encounter probability have one or more parameters that are related to home range size and movement rates of individuals about their home range. For example, the half-normal model above can be interpreted as implying a bivariate normal model for movement, where *σ*
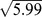
* is the 95% home range radius [14]. Detection of an individual at multiple traps provides information on σ, so we use the term “spatial captures” to refer to the number of unique traps at which an individual was detected or captured.

**Figure 2:**
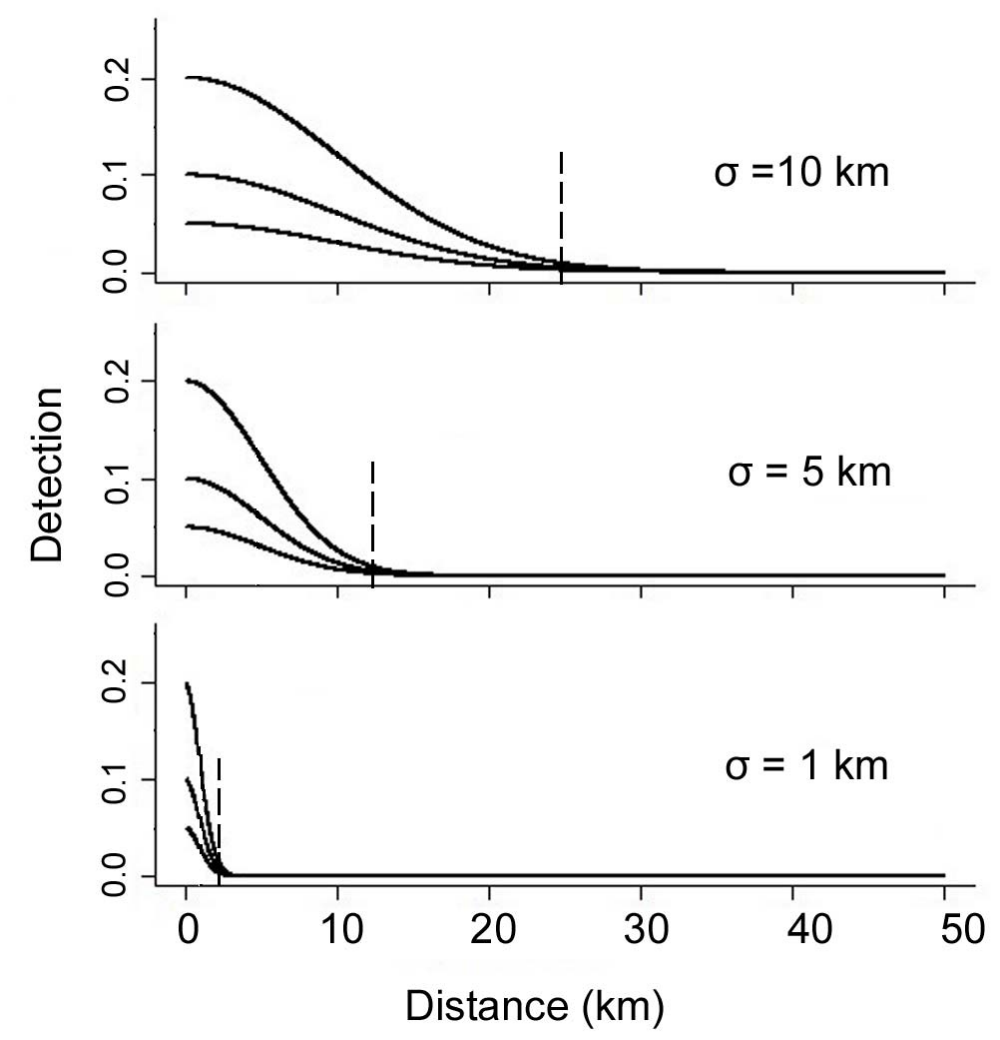
Nine detection scenarios by varying *σ* and *p_0_*. Nine detection scenarios for N = 500 were created by evaluating three values of the spatial scale parameter, (σ = 10, 5, and 1 km), for each of three baseline detection rates, (p_0_ = 0.20, 0.10, 0.05). As distance from an individual’s activity center increases, detection decreases according to a half-normal function based on the two parameters. Dashed vertical lines indicate 95% home range radii (σ*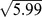).

We simulated SCR data for a population size of N = 500 over K = 10 sampling occasions. We distributed individuals over the 4,100 km^2^ landscape according to a random, uniform distribution, allowing for overlapping home ranges. This translates to a black bear density of 12.2 bears/100 km^2^, which is in the middle range of bear densities across the United States (Snowy Range of southeast Wyoming = 2.54 bears/100 km^2^) [29], central Appalachian Mountains in Kentucky = 8 bears/100 km^2^ [30], northern New York = 20 bears/100 km^2^ [8], north-central Pennsylvania = 23 bears/100 km^2^ [27], and Great Smoky Mountains, Tennessee ≥29 bears/100 km^2^ [31]. We created nine detection scenarios by varying the spatial scale parameter, σ, and the baseline detection probability, *p_0_* (Figure 2). We used three values of σ (1, 5, and 10 km) to model a range of representative home range sizes, spanning estimates of female and male home ranges typical of bears in the northeastern United States [8], [27], [32], [33]. We used three values of *p_0_* (0.05, 0.10, and 0.20) to explore a realistic range for mark-recapture studies. The upper limit, *p_0_* = 0.20 (i.e., 20%), is the minimum suggested detection probability in non-spatial mark-recapture studies [34], [35], but lower probabilities have been found to be sufficient for populations larger than N>200 [31], so we also included lower detection probabilities.

### Objectives

**Figure 3:**
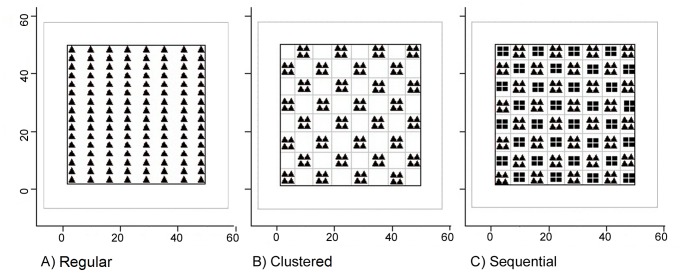
Three trap configurations: regular, clustered, and sequential. Three trap configurations were evaluated, shown with J = 128 traps: (a) regular array, (b), clustered, and (c) a temporal sequence in which clustered traps of one arrangement (e.g. triangle) are moved halfway through the sampling period to new grids (e.g. squares). Gray gridlines in (b) and (c) overlay the non-overlapping grid sizes of an estimated female home range. The black outline around the traps depicts the 2,624 km^2^ study area; the large gray square shows the extent of the 4,100 km^2^ landscape.

To evaluate the effect of sampling design on SCR parameter estimation, we applied three trap configurations: 1) regularly distributed across the study area, 2) grouped into clusters of 4 in every other non-overlapping female home range and, 3) traps relocated from one clustered configuration halfway through the sampling period to a second clustered configuration (Figure 3).

**Figure 4:**
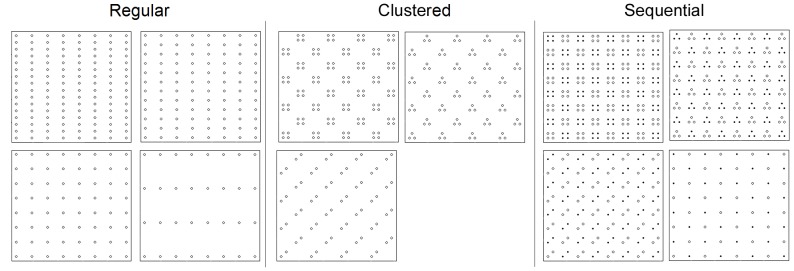
Trap configuration and number of traps generated eleven designs. Eleven trap designs were evaluated by varying the regular, clustered, and sequential trap arrangements for J = 128, 96, and 64 traps. Only the regular and sequential arrangements were evaluated for J = 32 traps since the clustered arrangement with one trap per cluster was equivalent to the regular arrangement. Trap spacing did not change when traps were in the clustered and sequential arrangements.

To evaluate trap spacing over the study area, we increased trap spacing from 4.7 km to 9.6 km by decreasing the number of traps from J = 128 traps to 96, 64, and 32 traps over the same spatial extent in the regular trap configuration (Table 1, Figure 4). This also resulted in different effective trap spacings, trap spacings relative to each value of σ, ranging from 0.47σ, when σ = 10 km, to 9.60σ when σ = 1 km (Table 2). Decreasing the number of traps resulted in a trap density of 0.049/km^2^ with 128 traps, 0.037/km^2^ with 96 traps, 0.024/km^2^ with 64 traps, and 0.012/km^2^ with 32 traps. The upper limit of 128 traps represents what could be realistically employed over such a large study area given a sampling frequency of once per week assuming two field teams, while also maintaining a minimum of 4 trap sites per estimated female home range. However, even this upper bound of trap density falls severely short of suggestions for black bear studies of 0.17–0.50/km^2^ [29]. We decreased the number of traps for the clustered and sequential trap configurations, although this did not change trap spacing. We calculated trap spacing for the regular trap configuration as the distance between a trap and the next closest trap, or for the clustered and sequential trap configurations, the distance between the centroids of a cluster and the next cluster. We did not consider the clustered trap configuration when J = 32 since clusters would have consisted of only 1 trap and therefore be equivalent to the regular configuration.

**Table 1:** Trap spacing (km) for each combination of trap configuration (regular, clustered, and sequential) and number of traps (J = 128, 96, 64, and 32).

	Number of traps, J				
	128	96	64		32
**Regular**	4.71	5.24	6.4		9.6
**Clustered**	9.06	9.06	9.06		N/A
**Sequential**	9.06	9.06	9.06	9.06	

Trap spacing (km) in the regular trap configuration was varied by decreasing the number of traps in the study area. Trap spacing did not vary when traps were in the clustered or sequential configurations because reductions only decreased the number of traps per cluster.

**Table 2:** Effective trap spacings for each σ, scaled by dividing trap spacings (4.71, 5.24, 6.40, and 9.60 km) by σ (1, 5, 10 km).

	σ = 1 km						σ = 5 km				σ = 10 km			
	Trap spacing (km)						Trap spacing (km)				Trap spacing (km)			
	4.71		5.24		6.40	9.60	4.71	5.24	6.40	9.60	4.71	5.24	6.40	9.60
**Regular**	4.71		5.24		6.40	9.60	0.94	1.05	1.28	1.92	0.47	0.52	0.64	0.96
**Clustered**	9.06	9.06		9.06		N/A	1.81	1.81	1.81	N/A	0.91	0.91	0.91	N/A
**Sequential**	9.06	9.06		9.06		9.06	1.81	1.81	1.81	1.81	0.91	0.91	0.91	0.91

For example, a trap spacing of 4.71 km equals 4.71σ when σ = 1 km but only 0.47σ when σ = 10 km.

Trap spacing of 9.60 km was not evaluated for the clustered trap configuration because it employs J = 32 traps and therefore is equivalent to the regular trap spacing.

For each of the nine detection scenarios (p_0_ *x σ*), we generated 500 simulated encounter histories for each combination of trap configuration (n = 3) and trap spacing (n = 4). To estimate abundance, *N,* and the spatial scale parameter, *σ,* we used a maximum likelihood approach [36], [37]. We conducted the simulations using Program R [38] and custom-written scripts (Table S1 in File S1) with package ‘snowfall’ and ‘rlecuyer’ [39], [40]. Estimates of N and *σ* were compared to the simulated truth. We used estimated means, standard deviations, ranges, root mean squared error (RMSE), and mean normalized bias (MNB) to evaluate the effects of trap configuration and spacing.

## Results

### Trap Configurations

The clustered trap configuration generally resulted in the most accurate estimators of abundance, 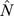. The clustered trap configuration yielded the lowest RMSEs in 8 of 9 combinations of p_0_ (3 cases) and σ (3 cases), i.e., with the exception of σ = 5 km and p_0_ = 0.20 in which the sequential trap configuration resulted in the most accurate 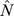 (Table 3). The three trap configurations resulted in similarly unbiased estimators of 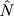 when effective trap spacing was <4.71σ, i.e., when σ >1 km. But when effective trap spacing ≥4.71σ (σ = 1 km), the clustered and sequential trap configurations resulted in the lowest MNBs in the remaining 1 and 2 cases, respectively.

**Table 3:** Summary estimates of 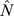 when true population size N = 500 and J = 128 traps, under each of the three trap arrangements: regular, clustered, and sequential, where mean, standard deviation (SD), range, root mean squared error (RMSE), and mean normalized bias (MNB) are given for each scenario (*p x σ x configuration)*.

	σ = 1 km						σ = 5 km						σ = 10 km					
p_0_ = 0.20	Mean	SD	Min	Max	RMSE	MNB	Mean	SD	Min	Max	RMSE	MNB	Mean	SD	Min	Max	RMSE	MNB
**Regular**	509.0	75.1	323.7	843.7	75.57	0.00	499.9	6.4	482.0	518.3	6.38	0.00	499.9	0.3	498.0	500.0	0.31	0.00
**Clustered**	503.4	60.8	344.8	683.6	60.80	0.01	499.3	5.9	480.0	516.1	5.93	0.00	500.0	0.2	499.0	500.0	0.20	0.00
**Sequential**	508.4	65.7	328.0	769.6	66.20	0.00	499.8	5.6	479.2	514.1	5.58	0.00	499.9	0.2	499.0	500.0	0.24	0.00
**p_0_ = 0.10**																		
**Regular**	546.7	177.9	240.4	1696.0	183.78	−0.01	499.7	9.4	471.5	525.5	9.41	0.00	499.6	1.1	495.8	501.1	1.14	0.00
**Clustered**	513.1	112.9	293.3	1168.3	113.58	0.02	499.7	8.5	471.3	523.3	8.53	0.00	499.5	1.0	495.3	500.7	1.13	0.00
**Sequential**	541.6	143.2	236.0	1164.9	148.94	−0.02	499.7	8.8	472.3	524.9	8.81	0.00	499.4	1.0	496.2	500.6	1.14	0.00
**p_0_ = 0.05**																		
**Regular**	2.8E11	6.3E12	1.7E2	1.41E14	6.29E12	−0.23	499.2	14.1	454.3	538.3	14.08	0.00	499.6	3.0	491.3	507.2	3.04	0.00
**Clustered**	3.3E4	7.2E5	156.1	1.60E7	7.14E5	0.08	499.9	13.8	447.1	541.0	13.81	0.00	499.7	2.9	490.0	505.9	2.94	0.00
**Sequential**	7.0E4	1.5E6	126.4	3.43E7	1.54E6	0.02	500.6	14.0	449.0	537.9	13.97	0.00	499.3	3.0	482.3	506.3	3.12	0.00

Estimates are averages of 500 simulations.

As the number of detected individuals and captures per individual increased because of closer effective trap spacings, estimators of 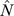 improved (Table S2 in File S1). For example, consider the clustered trap configuration when p_0_ = 0.05: when effective trap spacing decreased from 9.06σ to 0.91σ due to an increase in the fixed, biologically-determined σ from 1 km to 10 km, the number of detected individuals increased from 38 individuals with fewer than 2 spatial recaptures to 493 individuals (>98% of total population N = 500) with 7.8 spatial recaptures (Table S2 in File S1). When effective trap spacing was ≥4.71σ (i.e., when σ ≥1 km), the sparse datasets, especially low rates of spatial recaptures, resulted in unstable maximum likelihood estimators (MLEs) and a strongly right-skewed sampling distribution of 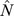 (Table 3). Therefore, population size was consistently overestimated at all trap arrangements and detection rates. Standard deviations (SD) and root mean square errors (RMSE) were both at least 12% (Table 3). However, when effective trap spacing was <4.71σ (i.e., when σ ≥5 km), the mean 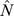 for all trap configurations at all detection probabilities were within one individual of the true N = 500, and SD and RMSE no more than 3% (Table 3).

Estimators of 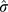 performed similarly well across the three trap configurations (Table 4). However, precision of the estimators for any given trap configuration increased when the effective trap spacing increased with larger values of σ. Comparing estimators across regular, clustered, and sequential trap configurations when effective trap spacings were ≥4.71σ and ≤0.91σ (i.e., σ = 1 km versus σ = 10 km), SD decreased from a maximum of 28% to 1.1% while MNB also decreased from a maximum of 18% to 0.2% (Table 4).

**Table 4:** Summary estimates of 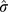 when the true population size N = 500 and J = 128 traps, under each of the three trap arrangements: regular, clustered, and sequential, where mean, standard deviation (SD), range, root mean squared error (RMSE), and mean normalized bias (MNB) are given for each scenario (*p x σ x configuration)*.

	σ = 1 km						σ = 5 km						σ = 10 km					
p_0_ = 0.20	Mean	SD	Min	Max	RMSE	MNB	Mean	SD	Min	Max	RMSE	MNB	Mean	SD	Min	Max	RMSE	MNB
**Regular**	1.00	0.07	0.80	1.22	0.07	−0.01	5.00	0.04	4.89	5.16	0.04	0.01	9.99	0.06	9.84	10.17	0.06	0.02
**Clustered**	1.00	0.07	0.81	1.27	0.07	−0.01	5.00	0.04	4.85	5.11	0.04	0.01	10.00	0.05	9.81	10.16	0.05	0.01
**Sequential**	1.00	0.08	0.76	1.25	0.08	−0.01	5.00	0.04	4.87	5.13	0.04	0.00	10.00	0.05	9.84	10.17	0.05	0.00
**p_0_ = 0.10**																		
**Regular**	1.00	0.13	0.57	1.41	0.13	−0.03	5.00	0.06	4.86	5.31	0.06	0.00	9.99	0.08	9.74	10.24	0.08	0.02
**Clustered**	1.01	0.14	0.63	1.52	0.15	−0.05	5.00	0.07	4.77	5.22	0.07	0.01	10.00	0.08	9.74	10.21	0.08	0.00
**Sequential**	0.99	0.14	0.67	1.45	0.14	−0.03	5.00	0.07	4.83	5.20	0.07	0.00	10.00	0.07	9.80	10.26	0.07	−0.01
**p_0_ = 0.05**																		
**Regular**	0.97	0.24	0.38	1.89	0.24	−0.05	5.00	0.10	4.73	5.34	0.10	0.00	10.00	0.11	9.68	10.40	0.11	0.01
**Clustered**	1.06	0.28	0.59	1.98	0.28	−0.18	4.99	0.10	4.66	5.47	0.10	0.02	10.00	0.11	9.58	10.33	0.11	0.01
**Sequential**	1.01	0.24	0.55	2.44	0.24	−0.10	5.00	0.10	4.68	5.28	0.10	0.00	10.00	0.11	9.67	10.44	0.11	−0.01

Estimates are averages of 500 simulations.

### Trap Spacings and Traps per Cluster

As trap spacing increased from 4.71 km to 9.60 km by reducing the number of traps (J = 128 to 32 traps), effective trap spacing relative to σ increased (Table 2). Individuals were detected fewer times and with fewer spatial and non-spatial captures (Table S3 in File S1). As a result, estimators of 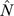 and 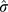 decreased in accuracy and precision as trap spacing increased and number of traps per cluster decreased (Table 5,6 and Tables S5–9 in File S1). For example, consider increased effective trap spacing from 4.71σ to 9.60σ (when σ = 1 km) at p_0_ = 0.20: population size was increasingly overestimated as the number of detected individuals decreased 73% and the spatial captures decreased from 1.1 to 1.0 (Table S3 in File S1). 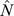 increased from 509 to 637, RMSE increased from 15 to 84% (regular trap configuration, Table 5), and RMSE of 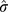 increased from 7% to 24% (Table 6). In some cases, including all trap spacings and trap configurations when p_0_ = 0.05, the number of detected individuals was as low as 10 individuals (2% of total population N = 500) and some simulated datasets yielded only one capture for all detected individuals (Table S3 in File S1). These sparse data sets caused the MLE to occur on the boundary of the parameter space, and simulated data sets for which this was the case were removed from the analysis. For example, 231 such cases were discarded under the sequential trap arrangement when p_0_ = 0.05 (Table S4 in File S1).

**Table 5:** For σ = 1 km, summary estimates of 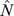 in the regular trap configuration when trap spacing increased from 4.71 to 9.60 km (J = 128 to 32 traps) and N = 500.

p_0_ = 0.20	Mean	SD	Min	Max	RMSE	MNB
**4.71**	509.0	75.1	323.7	843.7	75.57	0.00
**5.24**	654.3	318.6	222.9	2059.0	353.73	−0.07
**6.4**	591.2	270.7	219.0	2111.5	285.41	0.00
**9.6**	*636.8*	*398.3*	*119.7*	*2744.5*	*419.88*	*0.08*
**p_0_ = 0.10**						
**4.71**	546.8	177.9	240.4	1696.0	183.78	−0.01
**5.24**	654.3	318.6	222.9	2059.0	353.73	−0.07
**6.4**	705.0	534.0	131.8	5726.3	571.49	0.04
**9.6**	*1.3E11*	*2.8E12*	*103.0*	*6.3E13*	*2.82E12*	*0.08*
**p_0_ = 0.05**						
**4.71**	*2.8E11*	*6.3E12*	*168.5*	*1.4E14*	*6.29E12*	*−0.02*
**5.24**	*1.3E7*	*2.9E8*	*120.2*	*6.5E9*	*2.90E8*	*−0.12*
**6.4**	*1.9E13*	*4.1E14*	*91.1*	*8.8E15*	*3.94E14*	*0.21*
**9.6**	*3.1E11*	*5.0E12*	*45.0*	*9.7E13*	*4.39E12*	*0.35*

<500 iterations were used for the italicized estimates, due to instability of MLE with sparse datasets.

At p_0_ = 0.20 and trap spacing of 9.60 km, 498 iterations were used to calculate the mean estimate (2 iterations discarded).

At p_0_ = 0.10 and trap spacing of 9.60 km, 492 iterations were used to calculate the mean estimate (8 iterations discarded).

At p_0_ = 0.05, and trap spacings increasing from 4.71 km to 9.60 km, 497, 489, 457, and 381 iterations were used to calculate mean estimates (3, 11, 43, and 119 iterations discarded, respectively).

**Table 6:** For σ = 1 km, summary estimates of 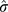 in the regular trap configuration when trap spacing increased from 4.71 to 9.60 km (J = 128 to 32 traps) and N = 500.

p_0_ = 0.20	Mean	SD	Min	Max	RMSE	MNB
**4.71**	1.00	0.07	0.80	1.22	0.07	−0.01
**5.24**	0.98	0.10	0.57	1.30	0.10	0.00
**6.40**	0.98	0.17	0.49	1.39	0.17	−0.03
**9.60**	*0.99*	*0.24*	*0.46*	*1.78*	*0.24*	*−0.08*
**p_0_ = 0.10**						
**4.71**	1.00	0.13	0.57	1.41	0.13	−0.03
**5.24**	0.96	0.19	0.50	1.76	0.20	−0.01
**6.40**	0.98	0.24	0.50	1.51	0.24	−0.07
**9.60**	*1.06*	*0.90*	*0.36*	*16.53*	*0.89*	*−0.33*
**p_0_ = 0.05**						
**4.71**	*0.97*	*0.24*	*0.38*	*1.89*	*0.24*	*−0.05*
**5.24**	*0.93*	*0.36*	*0.36*	*5.56*	*0.36*	*−0.04*
**6.40**	*1.02*	*0.32*	*0.39*	*2.90*	*0.31*	*−0.13*
**9.60**	*1.45*	*2.44*	*0.26*	*19.77*	*2.16*	*−1.26*

<500 iterations were used for the italicized estimates, due to instability of MLE with sparse datasets.

See Table 5 footnote for number of iterations used for the italicized estimates.

However, when effective trap spacing was ≤1.92σ (i.e., when σ = 5 and 10 km), the properties of the estimators 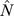 and 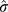 became similar across trap spacing and number of traps per cluster (Tables S5–9 in File S1). Estimators also increased in precision and accuracy. When σ = 10 km (p_0_ = 0.20), even as effective trap spacing increased from 0.47σ to 0.91σ, the number of detected individuals did not drop below 490 (98% of the true population N = 500,) until effective trap spacing decreased to 0.96σ when p_0_ = 0.10 and 0.52σ when p_0_ = 0.05 (Table S3 in File S1). As a result, estimators of 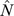 at all trap spacings were within 1 individual of the true population (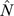 = 499.4 to 499.9) and RMSE was less than 1% (Table S6 in File S1). Estimators of 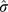 had RMSEs of less than 0.02% (Table S9 in File S1).

## Discussion

We demonstrated that the clustered trap configuration generally yielded the most accurate estimators of abundance, 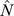. The regular trap configuration never out-performed the clustered or sequential trap arrangements in precision of abundance estimates, and in fact often resulted in fewer detected individuals, fewer total captures, and fewer spatial recaptures. Consequently, clustered and sequential trap arrangements even with fewer traps yielded estimates of abundance that were as precise or more as the regular trap configuration. Performance differences between the three trap configurations were most marked when trap spacing was large relative to home range size (Table 7). However, performance differences between trap configurations diminished as home range size increased.

**Table 7:** RMSE values of estimators of 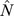, as effective trap spacing (i.e., trap spacing/σ) increased under the regular trap configuration and across all baseline detection probabilities (p_0_ = 0.20, 0.10, 0.05).

Trap spacing (σ)	p_0_ = 0.20	p_0_ = 0.10	p_0_ = 0.05
**0.47**	0.3	1.1	3
**0.52**	0.6	1.9	4.3
**0.64**	1.1	2.8	6.4
**0.94**	6.4	9.4	14.1
**0.96**	3.1	6.8	12.7
**1.05**	7.3	10.7	17.5
**1.28**	8.8	13.3	23.8
**1.92**	14.6	23.5	49.9
**4.71**	75.57	183.78	6.29E+12
**5.24**	353.73	353.73	2.90E+08
**6.4**	285.41	571.49	3.94E+14
**9.6**	419.88	2.82E+12	4.39E+12

SCR models are flexible to estimate population parameters with accuracy and precision for sampling designs commonly employed in studies of wide-ranging species. However, effective estimation in SCR models depends on obtaining a sufficiently large sample size of unique individuals and spatial recaptures. Compared to the regular trap configuration, the clustered arrangement frequently yielded more total captures and spatial recaptures, and the sequential arrangement yielded more unique individuals. Although the sequential configuration detected more unique individuals by moving traps to new locations, the total number of recaptures was fewer compared to the clustered configuration because each trap was only available to detect individuals for half the sampling occasions. Also, as detection rates decreased, more traps per cluster were necessary to detect individuals and recaptures. The necessity of sufficient sample sizes of individuals and spatial recaptures was also highlighted by the instability of the MLE under low detection (particularly p_0_ = 0.05) at small values of the spatial scale parameter (σ = 1 km), which resulted in parameter estimates on the boundary of the parameter space [41], [42].

Non-regular, and particularly the clustered, trap configurations helped compensate for sparse trap arrays. This suggests that precise estimates over a large study area are possible, even when limited by a sparse and widely-set trap array, by arranging traps in clusters. Clusters of traps increase the expected number of spatial recaptures of individuals while the large spatial extent increases the expected number of unique individuals detected.

Our simulations also suggest that it is important to prescribe trap spacing relative to home range sizes of individuals. As the spatial scale parameter, σ, increased, differences between the performance of SCR estimators with different trap configurations diminished. For example, at the smallest value of σ (1 km), trap spacing in the regular configuration was 4.71 km, or >4σ; but as σ increased to 10 km, this same trap spacing equated to just 0.47σ (Table 2). As a result, differences between trap arrangements were negligible at σ = 10 km, even at the lowest detection rate (p_0_ = 0.05). When traps are widely spaced relative to σ, fewer captures and spatial recaptures are collected. Accordingly, parameter estimates improved markedly when σ increased from 1 km to 5 km and trap spacing decreased to less than 2σ (Table 7). Home range diameters of black bears in the geographic region on which these simulations were based range from 5.1–25.1 km [27], coinciding with the value of σ when accuracy and precision of our parameter estimates improved. This pattern in trap spacing is similar to the conclusions of Sollmann et al. [15] that recommended trap distances be less than 2σ. Since σ is a spatial scale parameter related to an individual’s home range radius, this essentially suggests that at least ∼2 traps should be placed within an individual’s home range, a minimum that is smaller than the traditional recommendation for trap density of 4 traps per home range [17]. In evaluating trap spacings and configurations over a range of values for σ, our simulations also demonstrate the importance of establishing a sampling design based on the smallest (usually the female) estimate of σ. Doing so helps ensure detection of all individuals, even those with larger ranges of movement.

In field studies, implementing the sequential trap configuration requires that twice the number of traps be set because traps are moved half-way through the sampling period, which increases the amount of associated work that setting traps entails. Our simulations suggested that the different trap configurations performed similarly when trap spacing was less than 2σ, even when the sequential trap configuration detected a greater number of unique individuals. Thus, clustered trap configurations and even regular trap configurations may be sufficient, and more intense sampling designs unnecessary, when traps arrays with spacing of less than 2σ can be achieved. However, if trap spacing is >2σ, such as when forced due to large spatial extents, non-regular trap arrangements should be favored in order to maintain the precision of estimators. In this situation, our results suggest that the clustered configuration would likely be the most efficient to employ.

We identified several instances of tradeoff between precision (SD) and bias (MNB) in parameter estimation. However, values of RMSE, which incorporates bias and variance, were similar to the corresponding values of SD, and estimates of bias were low. Thus, any observed tradeoffs between precision and bias were not consequential.

Naturally, our simulations were not exhaustive of the parameter space. Particularly, we held the spatial extent constant to mimic conditions for a predetermined study area, and defined the upper bound of number of traps (J = 128) based on limits we expect researchers would likely face. As a result, we did not explore trap clusters with >4 traps, which would have allowed larger spatial extents by setting clusters farther apart than 9.06 km. Larger spatial extents allow for more individuals to be detected, and would be applicable for populations with lower densities and/or larger ranges of movement. At the same time, spatial extents smaller than examined here would provide further insight into the minimum requirements for robust parameter estimation. Such simulations that continue to investigate the balance between spatial extent and trap spacing would be valuable for future research.

## Conclusion

Our simulations demonstrate that 1) gains in precision and accuracy of parameter estimates are related to both trap configuration and trap spacing, which is relative to the spatial scale parameter and home range size, and that 2) increased numbers of traps per cluster (at least up to four traps per cluster) improve precision. Our simulations reinforce the understanding that although different SCR sampling designs can provide accurate and precise estimators of population parameters, effective estimation requires datasets that include captures and spatial recaptures of a sufficient proportion of the population. These results highlight the importance of understanding the spatial characteristics of a study population, such as home range sizes of different portions of the population, spatial scales of movement, as well as information about the ability to detect individuals.

In developing sampling designs for spatial capture-recapture studies, our results suggest the following strategy for devising a sampling design: 1) determine the spatial extent of the study population, 2) determine the maximum trap spacing based on the minimum value of the spatial scale parameter, 2σ_min_, 3) if enough traps are available to space traps less than 2σ in a regular arrangement, do so, assuming it is practical to implement, 4) otherwise, consider traps in a clustered configuration with wider spacing between clusters, and more traps per cluster as expected detection rate decreases. With the increasing application of SCR methods and the effort required of mark-recapture efforts, it is important to understand the consequences of different sampling designs for large-mammal populations. Simulations provide an accessible opportunity to explore different sampling arrangements, allowing researchers to identify feasible designs that most efficiently utilize effort and resources.

## Supporting Information

File S1 **Combined supporting information file containing Tables S1–S9.** Table S1 Custom-written R scripts for data simulation and parameter estimation. Table S2 in File S1. Summary of mean capture data across trap configuration, σ, and p_0_ for N = 500 and J = 128 traps. Table S3 in File S1. Summary of capture data across σ and p_0_ when trap spacing increased (4.71, 5.24, 6.40, and 9.60 km). Table S4 in File S1. For σ = 1 km, summary of estimated in the clustered and sequential trap configurations when trap spacing increased from 4.71 to 9.60 km (J = 128 to 32 traps) and N = 500. Table S5 in File S1. For σ = 5 km, summary estimates of in the regular, clustered, and sequential trap configurations when trap spacing increased from 4.71 to 9.60 km (J = 128 to 32 traps) and N = 500. Table S6 in File S1. For σ = 10 km, summary estimates of in the regular, clustered, and sequential trap configurations when trap spacing increased from 4.71 to 9.60 km (J = 128 to 32 traps) and N = 500. Table S7 in File S1. For σ = 1 km, summary of estimates of in the regular, clustered and sequential trap configurations when trap spacing increased from 4.71 to 9.60 km (J = 128 to 32 traps) and N = 500. Table S8 in File S1. For σ = 5 km, summary of estimates of in the regular, clustered and sequential trap configurations when trap spacing increased from 4.71 to 9.60 km (J = 128 to 32 traps) and N = 500. Table S9 in File S1. For σ = 10 km, summary of estimates of in the regular, clustered and sequential trap configurations when trap spacing increased from 4.71 to 9.60 km (J = 128 to 32 traps) and N = 500.
(DOC)
