# Correction: Trap Configuration and Spacing Influences Parameter Estimates in Spatial Capture-Recapture Models

**DOI:** 10.1371/journal.pone.0141634

**Published:** 2015-10-23

**Authors:** Catherine C. Sun, Angela K. Fuller, J. Andrew Royle

The authors would like to provide corrections to several of the values reported in this paper. Specifically, throughout the text, tables, and Supporting Information, the trap spacing should be 10.67 km instead of 9.60 km, due to correcting the spatial extent in the y-direction of the configurations with J = 32, necessitating re-simulations for J = 32. The authors have also recalculated the mean normalized bias (MNB). Revisions due to these changes can be found in Tables 1, 2, 3, 4, 5, 6, and 7 below, as well as sections B-I in Supporting Information file S1 File.

Please see S1 Appendix for descriptions of additional, supporting simulations.

**Table 1:** Trap spacing (km) for each combination of trap configuration (regular, clustered, and sequential) and number of traps (J = 128, 96, 64, and 32)

	Number of traps, J			
	128	96	64	32
**Regular**	4.71	5.24	6.4	10.67
**Clustered**	9.06	9.06	9.06	N/A
**Sequential**	9.06	9.06	9.06	9.06

Trap spacing (km) in the regular trap configuration was varied by decreasing the number of traps in the study area. Trap spacing did not vary when traps were in the clustered or sequential configurations because reductions only decreased the number of traps per cluster.

**Table 2:** Effective trap spacings for each σ, scaled by dividing trap spacings (4.71, 5.24, 6.40, and 10.67 km) by σ (1, 5, 10 km).

	σ = 1 km				σ = 5 km				σ = 10 km			
	Trap spacing (km)				Trap spacing (km)				Trap spacing (km)			
	**4.71**	**5.24**	**6.40**	**10.67**	**4.71**	**5.24**	**6.40**	**10.67**	**4.71**	**5.24**	**6.40**	**10.67**
**Regular**	4.71	5.24	6.40	10.67	0.94	1.05	1.28	2.10	0.47	0.52	0.64	1.07
**Clustered**	9.06	9.06	9.06	N/A	1.81	1.81	1.81	N/A	0.91	0.91	0.91	N/A
**Sequential**	9.06	9.06	9.06	9.06	1.81	1.81	1.81	1.81	0.91	0.91	0.91	0.91

For example, a trap spacing of 4.71km equals 4.71σ when σ = 1 km but only 0.47σ when σ = 10 km.

Trap spacing of 10.67 km was not evaluated for the clustered trap configuration because it employs J = 32 traps and therefore is equivalent to the regular trap spacing.

**Table 3:** Summary estimates of $\hat{N}$ when true population size N = 500 and J = 128 traps, under each of the three trap arrangements: regular, clustered, and sequential, where mean, standard deviation (SD), range, root mean squared error (RMSE), and mean normalized bias (MNB) are given for each scenario (*p x σ x configuration)*.

	σ = 1 km						σ = 5 km						σ = 10 km					
p_0_ = 0.20	Mean	SD	Min	Max	RMSE	MNB	Mean	SD	Min	Max	RMSE	MNB	Mean	SD	Min	Max	RMSE	MNB
**Regular**	509.0	75.1	323.7	843.7	75.57	1.8	499.9	6.4	482.0	518.3	6.38	0.00	499.9	0.3	498.0	500.0	0.31	0.00
**Clustered**	503.4	60.8	344.8	683.6	60.80	0.7	499.3	5.9	480.0	516.1	5.93	-0.10	500.0	0.2	499.0	500.0	0.20	0.00
**Sequential**	508.4	65.7	328.0	769.6	66.20	1.7	499.8	5.6	479.2	514.1	5.58	0.00	499.9	0.2	499.0	500.0	0.24	0.00
**p**_**0**_ **= 0.10**																		
**Regular**	546.7	177.9	240.4	1696.0	183.78	9.3	499.7	9.4	471.5	525.5	9.41	-0.10	499.6	1.1	495.8	501.1	1.14	-0.10
**Clustered**	513.1	112.9	293.3	1168.3	113.58	2.6	499.7	8.5	471.3	523.3	8.53	-0.10	499.5	1.0	495.3	500.7	1.13	-0.10
**Sequential**	541.6	143.2	236.0	1164.9	148.94	8.3	499.7	8.8	472.3	524.9	8.81	-0.10	499.4	1.0	496.2	500.6	1.14	-0.10
**p**_**0**_ **= 0.05**																		
**Regular**	*684*.*0*	*473*.*0*	*168*.*5*	*3735*.*5*	*507*.*1*	*36*.*8*	499.2	14.1	454.3	538.3	14.08	-0.20	499.6	3.0	491.3	507.2	3.04	-0.10
**Clustered**	*572*.*2*	*354*.*1*	*156*.*1*	*4561*.*6*	*361*.*1*	*14*.*4*	499.9	13.8	447.1	541.0	13.81	0.00	499.7	2.9	490.0	505.9	2.94	-0.10
**Sequential**	*665*.*5*	*443*.*1*	*126*.*4*	*3649*.*3*	*472*.*6*	*33*.*1*	500.6	14.0	449.0	537.9	13.97	0.10	499.3	3.0	482.3	506.3	3.12	-0.10

<500 iterations were used for the italicized estimates, due to the instability of MLE with sparse datasets. At p_0_ = 0.05 and σ = 1 km, 496, 498, and 493 iterations were used for the Regular, Clustered, and Sequential configurations, respectively. (4, 2, and 7 iterations were discarded).

**Table 4:** Summary estimates of $\hat{\sigma }$ when the true population size N = 500 and J = 128 traps, under each of the three trap arrangements: regular, clustered, and sequential, where mean, standard deviation (SD), range, root mean squared error (RMSE), and mean normalized bias (MNB) are given for each scenario (*p x σ x configuration)*.

	σ = 1 km						σ = 5 km						σ = 10 km					
p_0_ = 0.20	Mean	SD	Min	Max	RMSE	MNB	Mean	SD	Min	Max	RMSE	MNB	Mean	SD	Min	Max	RMSE	MNB
**Regular**	1.00	0.07	0.80	1.22	0.07	0.35	5.00	0.04	4.89	5.16	0.04	-0.04	9.99	0.06	9.84	10.17	0.06	-0.06
**Clustered**	1.00	0.07	0.81	1.27	0.07	0.02	5.00	0.04	4.85	5.11	0.04	-0.07	10.00	0.05	9.81	10.16	0.05	-0.03
**Sequential**	1.00	0.08	0.76	1.25	0.08	-0.17	5.00	0.04	4.87	5.13	0.04	-0.02	10.00	0.05	9.84	10.17	0.05	0.01
**p**_**0**_ **= 0.10**																		
**Regular**	1.00	0.13	0.57	1.41	0.13	0.12	5.00	0.06	4.86	5.31	0.06	0.02	9.99	0.08	9.74	10.24	0.08	-0.07
**Clustered**	1.01	0.14	0.63	1.52	0.15	1.22	5.00	0.07	4.77	5.22	0.07	-0.06	10.00	0.08	9.74	10.21	0.08	0.00
**Sequential**	0.99	0.14	0.67	1.45	0.14	-0.81	5.00	0.07	4.83	5.20	0.07	-0.02	10.00	0.07	9.80	10.26	0.07	0.03
**p**_**0**_ **= 0.05**																		
**Regular**	0.97	0.24	0.38	1.89	0.24	-3.30	5.00	0.10	4.73	5.34	0.10	0.00	10.00	0.00	9.68	10.40	0.11	-0.03
**Clustered**	1.06	0.28	0.59	1.98	0.28	5.91	4.99	0.10	4.66	5.47	0.10	0.02	10.00	-0.15	9.58	10.33	0.11	-0.03
**Sequential**	1.01	0.24	0.55	2.44	0.24	-0.10	5.00	0.10	4.68	0.62	0.10	0.00	10.00	-0.04	9.67	10.44	0.11	0.02

<500 iterations were used for the italicized estimates, due to the instability of MLE with sparse datasets.

At p_0_ = 0.05 and σ = 1 km, 496, 498, and 493 iterations were used for the Regular, Clustered, and Sequential configurations, respectively. (4, 2, and 7 iterations were discarded).

**Table 5:** For σ = 1 km, summary estimates of $\hat{N}$ in the regular trap configuration when trap spacing increased from 4.71 to 10.67 km (J = 128 to 32 traps) and N = 500.

p_0_ = 0.20	Mean	SD	Min	Max	RMSE	MNB
**4.71**	509.0	75.1	323.7	843.7	75.57	1.80
**5.24**	551.5	159.2	269.6	1557.6	167.20	10.29
**6.4**	591.2	270.7	219.0	2111.5	285.41	18.25
**10.67**	690.2	458.5	168.8	2761.9	495.94	38.04
**p**_**0**_ **= 0.10**						
**4.71**	546.8	177.9	240.4	1696.0	183.78	9.35
**5.24**	654.3	318.6	222.9	2059.0	353.73	30.86
**6.4**	705.0	534.0	131.8	5726.3	571.49	40.99
**10.67**	*918*.*0*	*907*.*6*	*82*.*0*	*11129*.*5*	*998*.*40*	*83*.*60*
**p**_**0**_ **= 0.05**						
**4.71**	*684*.*0*	*473*.*0*	*168*.*5*	*3735*.*5*	*507*.*07*	*36*.*81*
**5.24**	*914*.*2*	*738*.*2*	*120*.*2*	*5739*.*5*	*845*.*84*	*82*.*84*
**6.4**	*755*.*6*	*746*.*9*	*91*.*1*	*4637*.*3*	*788*.*63*	*51*.*12*
**10.67**	*660*.*1*	*627*.*9*	*54*.*0*	*3238*.*4*	*647*.*10*	*32*.*02*

<500 iterations were used for the italicized estimates, due to instability of MLE with sparse datasets.

At p_0_ = 0.10 and trap spacing of 10.67 km, 488 iterations were used to calculate the mean estimate (12 iterations discarded).

At p_0_ = 0.05, and trap spacings increasing from 4.71km to 10.67 km, 496, 488, 447, and 328 iterations were used to calculate mean estimates (4, 12, 53, and 172 iterations discarded, respectively).

**Table 6:** For σ = 1 km, summary estimates of $\hat{\sigma }$ in the regular trap configuration when trap spacing increased from 4.71 to 10.67 km (J = 128 to 32 traps) and N = 500.

p_0_ = 0.20	Mean	SD	Min	Max	RMSE	MNB
**4.71**	1.00	0.07	0.80	1.22	0.07	0.35
**5.24**	0.98	0.10	0.57	1.30	0.10	-1.77
**6.40**	0.98	0.17	0.49	1.39	0.17	-1.79
**10.67**	0.97	0.27	0.41	1.47	0.27	-2.73
**p**_**0**_ **= 0.10**						
**4.71**	1.00	0.13	0.57	1.41	0.13	0.12
**5.24**	0.96	0.19	0.50	1.76	0.20	-4.17
**6.40**	0.98	0.24	0.50	1.51	0.24	-1.72
**10.67**	*0*.*93*	*0*.*34*	*0*.*34*	*1*.*85*	*0*.*34*	*-7*.*02*
**p**_**0**_ **= 0.05**						
**4.71**	*0*.*97*	*0*.*24*	*0*.*38*	*1*.*89*	*0*.*24*	*-3*.*30*
**5.24**	*0*.*93*	*0*.*30*	*0*.*36*	*2*.*31*	*0*.*36*	*-7*.*51*
**6.40**	*1*.*02*	*0*.*31*	*0*.*39*	*1*.*77*	*0*.*31*	*1*.*57*
**10.67**	*0*.*99*	*2*.*41*	*0*.*23*	*2*.*02*	*2*.*16*	*-1*.*50*

<500 iterations were used for the italicized estimates, due to instability of MLE with sparse datasets.

See Table 5 footnote for number of iterations used for the italicized estimates.

**Table 7:** RMSE values of estimators of $\hat{N}$, as effective trap spacing (i.e., trap spacing/ σ) increased under the regular trap configuration and across all baseline detection probabilities (p_0_ = 0.20, 0.10, 0.05).

Trap spacing (σ)	σ (km)	J	p_0_ = 0.20	p_0_ = 0.10	p_0_ = 0.05
**0.47**	10	128	0.3	1.1	3.0
**0.52**	10	96	0.6	1.9	4.3
**0.64**	10	64	1.1	2.8	6.4
**1.07**	10	32	2.2	5.7	12.2
**0.94**	5	128	6.4	9.4	14.1
**1.05**	5	96	7.3	10.7	17.5
**1.28**	5	64	8.8	13.3	23.8
**2.13**	5	32	12.6	24.2	49.9
**4.71**	1	128	75.6	183.8	507.1
**5.24**	1	96	167.2	353.7	845.8
**6.4**	1	64	285.4	571.5	788.6
**10.67**	1	32	495.8	889.4	647.1

The authors have provided a corrected explanation of the calculation in the second paragraph of the “Objectives” subsection of the Methods here:

To evaluate trap spacing over the study area, we increased trap spacing from 4.7 km to 10.67 km by decreasing the number of traps from J = 128 traps to 96, 64, and 32 traps over the same spatial extent in the regular trap configuration (Table 1, Figure 4). This also resulted in different effective trap spacings, trap spacings relative to each value of σ, ranging from 0.47σ, when σ = 10 km, to 10.67σ when σ = 1 km (Table 2). Decreasing the number of traps resulted in a trap density of 0.049/km^2^ with 128 traps, 0.037/km^2^ with 96 traps, 0.024/km^2^ with 64 traps, and 0.012/km^2^ with 32 traps. The upper limit of 128 traps represents what could be realistically employed over such a large study area given a sampling frequency of once per week assuming two field teams, while also maintaining a minimum of 4 trap sites per estimated female home range. However, even this upper bound of trap density falls severely short of suggestions for black bear studies of 0.17–0.50/km^2^ [29]. We decreased the number of traps for the clustered and sequential trap configurations, although this did not change trap spacing. We calculated trap spacing for the regular trap configuration as the average distance between a trap and its 4 closest neighbors, or for the clustered and sequential trap configurations, the distance between a centroid of a cluster and the next cluster. We did not consider the clustered trap configuration when J = 32 since clusters would have consisted of only 1 trap and therefore be equivalent to the regular configuration.

The last sentence of the first paragraph of the “Trap configurations” subsection of the Results should read:

But when effective trap spacing ≥ 4.71σ (σ = 1 km), the clustered and trap configuration resulted in the lowest MNBs.

The last sentence of the third paragraph of the “Trap configurations” subsection of the Results should read:

Comparing estimators across regular, clustered, and sequential trap configurations when effective trap spacings were ≥4.71σ and ≤ 0.91σ (i.e., σ = 1 km versus σ = 10 km), SD decreased from a maximum of 28% to 1.1% while MNB also decreased from a maximum of 5.9% to 0.028% (Table 4).

The final two paragraph of the Results should read:

As trap spacing increased from 4.71 km to 10.67 km by reducing the number of traps (J = 128 to 32 traps), effective trap spacing relative to σ increased (Table 2). Individuals were detected fewer times and with fewer spatial and non-spatial captures (Table S3 in S1 File). As a result, estimators of $\hat{N}$ and $\hat{\sigma }$ decreased in accuracy and precision as trap spacing increased and number of traps per cluster decreased (Table 5,6 and Tables S5-9 in S1 File). For example, consider increased effective trap spacing from 4.71σ to 10.67σ (when σ = 1 km) at p_0_ = 0.20: population size was increasingly overestimated as the number of detected individuals decreased 73% and the spatial captures decreased from 1.1 to 1.0 (Table S3 in S1 File). $\hat{N}$ increased from 509 to 690, RMSE increased from 15 to 99% (regular trap configuration, Table 5), and RMSE of $\hat{\sigma }$ increased from 7% to 27% (Table 6). In some cases, including all trap spacings and trap configurations when p_0_ = 0.05, the number of detected individuals was as low as 40 individuals (8% of total population N = 500) and some simulated datasets yielded only one capture for all detected individuals (Table S3 in S1 File). These sparse data sets caused the MLE to occur on the boundary of the parameter space, and simulated data sets for which this was the case were removed from the analysis. For example, 308 such cases were discarded under the sequential trap arrangement when p_0_ = 0.05 (Table S4 in S1 File).

However, when effective trap spacing was ≤2.13σ (i.e., when σ = 5 and 10 km), the properties of the estimators $\hat{N}$ and $\hat{\sigma }$ became similar across trap spacing and number of traps per cluster (Tables S5-9 in S1 File). Estimators also increased in precision and accuracy. When σ = 10 km (p_0_ = 0.20), even as effective trap spacing increased from 0.47σ to 1.07σ, the number of detected individuals did not drop below 490 (98% of the true population N = 500,) until effective trap spacing decreased to 1.07σ when p_0_ = 0.10 and 0.52σ when p_0_ = 0.05 (Table S3 in S1 File). As a result, estimators of $\hat{N}$ at all trap spacings were within 1 individual of the true population ($\hat{N}$ = 499.4 to 500.1) and RMSE was less than 2.4% (Table S6 in S1 File). Estimators of $\hat{\sigma }$ had RMSEs of less than 1.7% (Table S9 in S1 File).

The fourth paragraph of the Discussion section has been revised for improved interpretation and should read:

Our simulations also suggest that it is important to prescribe trap spacing relative to home range sizes of individuals. As the spatial scale parameter, σ, increased, differences between the performance of SCR estimators with different trap configurations diminished. For example, at the smallest value of σ (1 km), trap spacing in the regular configuration was 4.71 km, or > 4σ; but as σ increased to 10 km, this same trap spacing equated to just 0.47σ (Table 2). As a result, differences between trap arrangements were negligible at σ = 10 km, even at the lowest detection rate (p_0_ = 0.05). When traps are widely spaced relative to σ, fewer captures and spatial recaptures are collected. Accordingly, parameter estimates improved markedly when σ increased from 1 km to 5 km and trap spacing decreased to less than 2σ (Table 7). The increase in σ from 1 km to 5 km corresponds to an increase in home range diameter from approximately 5 km to 12.2 km [2σ sqrt(5.99)]. This is consistent with an incrase from the minimum estimated home range diameter of 5.1 km [27] of black bears in the geographic region on which these simulations were based. This pattern in trap spacing is similar to the conclusions of Sollmann et al. and Efford and Fewster[15] that recommended trap distances be less than 2σ. Since σ is a spatial scale parameter related to an individual’s home range radius, this essentially suggests that at least ~2 traps should be placed within an individual’s home range, a minimum that is smaller than the traditional recommendation for trap density of 4 traps per home range [17]. In evaluating trap spacings and configurations over a range of values for σ, our simulations also demonstrate the importance of establishing a sampling design based on the smallest (usually the female) estimate of σ. Doing so helps ensure detection of all individuals, even those with larger ranges of movement.

## Supporting Information

S1 File Combined supporting information file containing Tables A-I. **A.** Custom-written R scripts for data simulation and parameter estimation. **B.** Summary of mean capture data across trap configuration, σ, and p_0_ for N = 500 and J = 128 traps. **C.** Summary of capture data across σ and p_0_ when trap spacing increased (4.71, 5.24, 6.40, and 10.67 km). **D.** For σ = 1 km, summary of estimated in the clustered and sequential trap configurations when trap spacing increased from 4.71 to 10.67 km (J = 128 to 32 traps) and N = 500. **E.** For σ = 5 km, summary estimates of in the regular, clustered, and sequential trap configurations when trap spacing increased from 4.71 to 10.67 km (J = 128 to 32 traps) and N = 500. **F.** For σ = 10 km, summary estimates of in the regular, clustered, and sequential trap configurations when trap spacing increased from 4.71 to 10.67 km (J = 128 to 32 traps) and N = 500. **G.** For σ = 1 km, summary of estimates of in the regular, clustered and sequential trap configurations when trap spacing increased from 4.71 to 10.67 km (J = 128 to 32 traps) and N = 500. **H.** For σ = 5 km, summary of estimates of in the regular, clustered and sequential trap configurations when trap spacing increased from 4.71 to 10.67 km (J = 128 to 32 traps) and N = 500. **I.** For σ = 10 km, summary of estimates of in the regular, clustered and sequential trap configurations when trap spacing increased from 4.71 to 10.67 km (J = 128 to 32 traps) and N = 500.
(DOC)

S1 Appendix Additional, supporting simulations. (DOCX)
