# Supplementary material for: Trap Configuration and Spacing Influences Parameter Estimates in Spatial Capture-Recapture Models
Source: PLoS One. 2014 Feb 5;9(2):e88025. doi: 10.1371/journal.pone.0088025 (PMC3914876; doi:10.1371/journal.pone.0088025)
Supplement: File S1 — Combined supporting information file containing Tables S1–S9. Table S1 Custom-written R scripts for data simulation and parameter estimation. Table S2 in File S1. Summary of mean capture data across trap configuration, σ, and p0 for N = 500 and J = 128 traps. Table S3 in File S1. Summary of capture data across σ and p0 when trap spacing increased (4.71, 5.24, 6.40, and 9.60 km). Table S4 in File S1. For σ = 1 km, summary of estimated in the clustered and sequential trap configurations when trap spacing increased from 4.71 to 9.60 km (J = 128 to 32 traps) and N = 500. Table S5 in File S1. For σ = 5 km, summary estimates of in the regular, clustered, and sequential trap configurations when trap spacing increased from 4.71 to 9.60 km (J = 128 to 32 traps) and N = 500. Table S6 in File S1. For σ = 10 km, summary estimates of in the regular, clustered, and sequential trap configurations when trap spacing increased from 4.71 to 9.60 km (J = 128 to 32 traps) and N = 500. Table S7 in File S1. For σ = 1 km, summary of estimates of in the regular, clustered and sequential trap configurations when trap spacing increased from 4.71 to 9.60 km (J = 128 to 32 traps) and N = 500. Table S8 in File S1. For σ = 5 km, summary of estimates of in the regular, clustered and sequential trap configurations when trap spacing increased from 4.71 to 9.60 km (J = 128 to 32 traps) and N = 500. Table S9 in File S1. For σ = 10 km, summary of estimates of in the regular, clustered and sequential trap configurations when trap spacing increased from 4.71 to 9.60 km (J = 128 to 32 traps) and N = 500. (DOC) [file pone.0088025.s001.doc]

**S1. Custom-written R scripts for data simulation and parameter estimation.**

#############

## functions ##

#############

# e2 dist:

e2dist <-

function (x, y)

{

i <- sort(rep(1:nrow(y), nrow(x)))

dvec <- sqrt((x[, 1] - y[i, 1])^2 + (x[, 2] - y[i, 2])^2)

matrix(dvec, nrow = nrow(x), ncol = nrow(y), byrow = F)

}

# intlik

intlik <-

function (start = NULL, y = y, K = NULL, delta = 0.3, X = traplocs,

G = NULL, ssbuffer = 2)

{

if (is.null(G)) {

Xl <- min(X[, 1]) - ssbuffer

Xu <- max(X[, 1]) + ssbuffer

Yu <- max(X[, 2]) + ssbuffer

Yl <- min(X[, 2]) - ssbuffer

SSarea <- (Xu - Xl) * (Yu - Yl)

if (is.null(K))

return("need sample size")

xg <- seq(Xl + delta/2, Xu - delta/2, delta)

yg <- seq(Yl + delta/2, Yu - delta/2, delta)

npix.x <- length(xg)

npix.y <- length(yg)

area <- (Xu - Xl) * (Yu - Yl)/((npix.x) * (npix.y))

G <- cbind(rep(xg, npix.y), sort(rep(yg, npix.x)))

}

else {

G <- G

SSarea <- nrow(G)

}

nG <- nrow(G)

D <- e2dist(X, G)

if (is.null(start))

start <- c(0, 0, 0)

alpha0 <- start[1]

alpha1 <- exp(start[2])

n0 <- exp(start[3])

probcap <- plogis(alpha0) * exp(-alpha1 * D * D)

Pm <- matrix(NA, nrow = nrow(probcap), ncol = ncol(probcap))

ymat <- y

ymat <- rbind(y, rep(0, ncol(y)))

lik.marg <- rep(NA, nrow(ymat))

for (i in 1:nrow(ymat)) {

Pm[1:length(Pm)] <- (dbinom(rep(ymat[i, ], nG), rep(K, nG),

probcap[1:length(Pm)], log = TRUE))

# when traps are relocation in the sequential arrangement

# Pm[1:length(Pm)] <- (dbinom(rep(ymat[i, ], nG), rep((K/2), nG),

probcap[1:length(Pm)], log = TRUE))

lik.cond <- exp(colSums(Pm))

lik.marg[i] <- sum(lik.cond * (1/nG))

}

nv <- c(rep(1, length(lik.marg) - 1), n0)

part1 <- lgamma(nrow(y) + n0 + 1) - lgamma(n0 + 1)

part2 <- sum(nv * log(lik.marg))

out <- -1 * (part1 + part2)

attr(out, "SSarea") <- SSarea

out

}

################################

# Simulate some number of datasets #

################################

Qfnmulti<-function(X,G,alpha0,sigma){

# Simulations

wrapper<-function(a){

nsim<-2

sims.mat <- matrix(NA,nsim,4)

colnames(sims.mat)<-c("alpha0","alpha1","N","Time")

set.seed(1234)

N <- 500 # N

K <- 10 # occasions

traplocs <- X

alpha0 <- alpha0

sigma <- sigma

for(sim in 1:nsim){

pickS <- sample(1:nrow(G),N,replace=T)

S <- G[pickS,]

D <- e2dist(S, traplocs)

ntraps <- nrow(traplocs)

# with trap relocation in the sequential arrangement

# traps_1<-traplocs[1:(nrow(traplocs)/2),] ; ntraps_1<-nrow(traps_1)

# traps_2<-traplocs[((nrow(traplocs)/2)+1):ntraps,] ; ntraps_2<-nrow(traps_2)

beta <- 1/(2 * sigma * sigma)

probcap <- plogis(alpha0) * exp(-beta * D * D)

Y <- matrix(NA, nrow = N, ncol = ntraps)

for (i in 1:nrow(Y)) {

Y[i,] <- rbinom(ntraps, K, probcap[i,])

}

# with trap relocation in the sequential arrangement

# for (i in 1:nrow(Y)) {

# Y[i,1:ntraps_1 ] <- rbinom(ntraps_1, K/2, probcap[i,1:ntraps_1 ])

# Y[i,(ntraps_1+1):ntraps ] <- rbinom(ntraps_2, K/2, probcap[i,((ntraps_1)+1):ntraps])

# }

totalcaps <- apply(Y, 1, sum)

Y <- Y[totalcaps > 0, ]

dimnames(Y) <- list(1:nrow(Y), paste("trap", 1:ncol(Y), sep = ""))

ninds<-nrow(Y)

hols <- list( Y = Y, traplocs = traplocs, N = N, alpha0 = alpha0,

beta = beta, sigma = sigma, K = K, S = S)

s3<-ifelse(is.infinite(log(N-nrow(Y))),log(100),log(N-nrow(Y)))

starts<-c(alpha0,log(beta),s3)

t <- system.time( out <- nlm(intlik, starts, hessian=TRUE, y=hols$Y,

K=hols$K, X=hols$traplocs, G = G))

sims.mat[sim,] <- c(out$estimate[1],out$estimate[2],nrow(hols$Y)+exp(out$estimate[3]),t[3])

}

return(sims.mat)

}

# library(“rlecuyer”)

# library(“snowfall’)

}

**S2. Summary of mean capture data across trap configuration, σ, and p_0_ for N=500 and J=128 traps.**

|  |  | **σ = 1 km** | | | **σ = 5 km** | | | **σ = 10 km** | | |
| --- | --- | --- | --- | --- | --- | --- | --- | --- | --- | --- |
| **p_0_** | **Design** | **Inds** | **Caps** | **Spatial Caps** | **Inds** | **Caps** | **Spatial Caps** | **Inds** | **Caps** | **Spatial Caps** |
| **0.20** | **Regular** | 113.0 | 1.6 | 1.1 | 460.8 | 10.0 | 6.6 | 499.9 | 33.7 | 22.2 |
|  | **Clustered** | 97.7 | 1.9 | 1.2 | 467.9 | 9.8 | 6.8 | 500.0 | 33.4 | 20.8 |
|  | **Sequential** | 128.4 | 1.4 | 1.2 | 470.7 | 9.7 | 8.4 | 499.9 | 33.4 | 27.2 |
| **0.10** | **Regular** | 70.6 | 1.3 | 1.1 | 425.2 | 5.4 | 4.4 | 498.8 | 16.8 | 13.7 |
|  | **Clustered** | 65.1 | 1.4 | 1.2 | 435.7 | 5.2 | 4.3 | 499.0 | 16.6 | 12.8 |
|  | **Sequential** | 75.4 | 1.2 | 1.0 | 437.1 | 5.2 | 6.8 | 499.0 | 16.6 | 14.7 |
| **0.05** | **Regular** | 39.9 | 1.1 | 1.1 | 372.2 | 3.1 | 2.7 | 491.1 | 8.5 | 7.7 |
|  | **Clustered** | 37.7 | 1.2 | 1.2 | 435.7 | 5.2 | 2.5 | 492.6 | 8.4 | 7.8 |
|  | **Sequential** | 40.8 | 1.1 | 1.0 | 383.7 | 2.9 | 6.7 | 492.4 | 8.4 | 7.7 |

p_0_ – baseline detection rate

Inds – average number of unique individuals detected

Caps – average number of total captures per individual

Spatial caps – average number of unique traps at which each individual was captured

Data are averages of 500 simulations.

**S3. Summary of capture data across σ and p_0_ when trap spacing increased (4.71, 5.24, 6.40, and 9.60 km)**

|  | **σ = 1 km** | | | **σ = 5 km** | | | **σ = 10 km** | | |
| --- | --- | --- | --- | --- | --- | --- | --- | --- | --- |
| **p_0_=0.20** | **Inds** | **Caps** | **Spatial Caps** | **Inds** | **Caps** | **Spatial Caps** | **Inds** | **Caps** | **Spatial Caps** |
| **4.71** | 113.0 | 1.6 | 1.1 | 460.8 | 10.0 | 6.6 | 499.9 | 33.7 | 22.2 |
| **5.24** | 89.0 | 1.6 | 1.0 | 450.1 | 7.6 | 5.3 | 499.7 | 25.2 | 17.1 |
| **6.4** | 60.8 | 1.5 | 1.0 | 435.9 | 5.3 | 3.7 | 499.1 | 16.7 | 11.3 |
| **9.6** | 30.2 | 1.5 | 1.0 | 371.8 | 3.1 | 2.1 | 490.4 | 8.5 | 5.6 |
| **p_0_=0.10** |  |  |  |  |  |  |  |  |  |
| **4.71** | 70.6 | 1.3 | 1.1 | 425.2 | 5.4 | 4.4 | 498.8 | 16.8 | 13.7 |
| **5.24** | 54.7 | 1.3 | 1.0 | 408.5 | 4.2 | 3.6 | 497.1 | 12.6 | 10.3 |
| **6.4** | 36.1 | 1.2 | 1.0 | 382.9 | 3.0 | 2.5 | 492.7 | 8.4 | 6.9 |
| **9.6** | 18.2 | 1.2 | 1.0 | 293.9 | 1.9 | 1.5 | 463.4 | 4.5 | 3.5 |
| **p_0_=0.05** |  |  |  |  |  |  |  |  |  |
| **4.71** | 39.9 | 1.1 | 1.1 | 372.2 | 3.1 | 2.7 | 491.1 | 8.5 | 7.7 |
| **5.24** | 30.8 | 1.1 | 1.0 | 344.4 | 2.5 | 2.3 | 483.8 | 6.5 | 5.9 |
| **6.4** | 20.2 | 1.1 | 1.0 | 299.4 | 1.9 | 1.7 | 466.8 | 4.4 | 4.1 |
| **9.6** | 10.0 | 1.1 | 1.0 | 198.5 | 1.4 | 1.3 | 400.1 | 2.6 | 2.3 |

Data are averages of 500 simulations.

**S4. For σ = 1 km, summary of estimated $\hat{N}$ in the clustered and sequential trap configurations when trap spacing increased from 4.71 to 9.60 km (J=128 to 32 traps) and N=500.**

|  | **Clustered** | | | | | | **Sequential** | | | | | |
| --- | --- | --- | --- | --- | --- | --- | --- | --- | --- | --- | --- | --- |
| **p_0_=0.20** | **Mean** | **SD** | **Min** | **Max** | **RMSE** | **MNB** | **Mean** | **SD** | **Min** | **Max** | **RMSE** | **MNB** |
| **4.71** | 503.4 | 60.8 | 344.8 | 683.6 | 60.8 | 0.0 | 508.4 | 65.7 | 328.0 | 769.6 | 66.2 | 0.0 |
| **5.24** | 508.5 | 89.0 | 273.1 | 835.3 | 89.3 | 0.0 | 516.0 | 90.9 | 315.6 | 959.4 | 92.2 | 0.0 |
| **6.40** | 534.2 | 135.3 | 248.0 | 1124.3 | 139.4 | 0.0 | 553.6 | 167.2 | 268.7 | 1553.9 | 175.4 | 0.0 |
| **9.60** | N/A | | | | | | *676.1* | *480.7* | *127.2* | *4945.2* | *510.9* | *0.1* |
| **p_0_=0.10** |  |  |  |  |  |  |  |  |  |  |  |  |
| **4.71** | 513.1 | 112.9 | 293.3 | 1168.3 | 113.6 | 0.0 | 541.6 | 143.2 | 236.0 | 1164.9 | 148.9 | 0.0 |
| **5.24** | 527.0 | 156.6 | 202.4 | 1195.1 | 158.8 | 0.0 | 581.7 | 246.5 | 242.0 | 1666.5 | 259.5 | 0.0 |
| **6.40** | 611.5 | 353.6 | 146.0 | 3938.6 | 370.4 | 0.0 | *814.5* | *664.6* | *160.6* | *5919.6* | *730.2* | *-0.1* |
| **9.60** | N/A | | | | | | *4.5E12* | *9.6E13* | *78.5* | *2.1E15* | *9.2E13* | *0.2* |
| **p_0_=0.05** |  |  |  |  |  |  |  |  |  |  |  |  |
| **4.71** | *3.3E4* | *72E5* | *156.1* | *1.6E7* | *7.1E5* | *0.1* | *7.0E4* | *1.5E6* | *126.4* | *3.4E7* | *1.5E6* | *0.0* |
| **5.24** | *1.7E6* | *2.7E7* | *113.9* | *5.5E8* | *2.6E7* | *0.2* | *2.2E11* | *4.7E12* | *98.0* | *1.0E14* | *4.5E12* | *0.1* |
| **6.40** | *1.5E8* | *3.2E9* | *67.8* | *6.9E10* | *3.1E9* | *0.2* | *5.2E14* | *7.8E15* | *71.9* | *1.4E17* | *6.8E15* | *0.2* |
| **9.60** | N/A | | | | | | *1.1E14* | *1.2E15* | *19.1* | *1.5E16* | *8.8E14* | *0.5* |

Clustered trap configurations were not evaluated at 9.60 trap spacing (J=32 traps) as it was equivalent to a Regular trap configuration.

<500 iterations were used for the italicized estimates, due to instability of MLE with sparse datasets.

Under the Clustered trap configuration at p_0_=0.05, and trap spacings increasing from 4.71km to 6.40km, 499, 497, and 473 iterations were used to calculate mean estimates (1,3, and 27 iterations discarded, respectively).

Under the Sequential trap configuration at p_0_=0.20 and trap spacing of 9.60 km, 499 iterations were used to calculate mean estimates (1 iteration discarded)

Under the Sequential trap configuration at p_0_=0.10 and trap spacings of 6.40km and 9.60km, 494 and 456 iterations were used to calculate mean estimates (6 and 44 iteration discarded, respectively).

Under the Sequential trap configuration at p_0_=0. 50 and trap spacing increasing from 4.17km to 9.60 km, 494,465,374 and 269 iterations were used to calculate mean estimates (6, 35, 126, and 231 iterations discarded, respectively)

**S5. For σ = 5 km, summary estimates of $\hat{N}$ in the regular, clustered, and sequential trap configurations when trap spacing increased** from 4.71 to 9.60 km (J=128 to 32 traps) and N=500.

|  | **Regular** | | | | | | **Clustered** | | | | | | **Sequential** | | | | | |
| --- | --- | --- | --- | --- | --- | --- | --- | --- | --- | --- | --- | --- | --- | --- | --- | --- | --- | --- |
| **p_0_=0.20** | **Mean** | **SD** | **Min** | **Max** | **RMSE** | **MNB** | **Mean** | **SD** | **Min** | **Max** | **RMSE** | **MNB** | **Mean** | **SD** | **Min** | **Max** | **RMSE** | **MNB** |
| **4.71** | 499.9 | 6.4 | 482.0 | 518.3 | 6.4 | 0.0 | 499.3 | 5.9 | 480.0 | 516.1 | 5.9 | 0.0 | 499.8 | 5.6 | 479.2 | 514.1 | 5.6 | 0.0 |
| **5.24** | 499.7 | 7.3 | 477.3 | 520.8 | 7.3 | 0.0 | 499.6 | 6.8 | 478.3 | 520.9 | 6.8 | 0.0 | 499.3 | 7.1 | 476.6 | 524.8 | 7.1 | 0.0 |
| **6.40** | 499.0 | 8.8 | 471.8 | 523.1 | 8.8 | 0.0 | 499.9 | 9.0 | 474.0 | 527.2 | 9.0 | 0.0 | 499.3 | 8.8 | 465.5 | 525.8 | 8.8 | 0.0 |
| **9.60** | 499.6 | 14.6 | 448.1 | 541.5 | 14.6 | 0.0 | N/A | | | | | | 498.8 | 13.3 | 456.9 | 537.5 | 13.3 | 0.0 |
| **p_0_=0.10** |  |  |  |  |  |  |  |  |  |  |  |  |  |  |  |  |  |  |
| **4.71** | 499.7 | 9.4 | 471.5 | 525.5 | 9.4 | 0.0 | 499.7 | 8.5 | 471.3 | 523.3 | 8.5 | 0.0 | 499.7 | 8.8 | 472.3 | 524.9 | 8.8 | 0.0 |
| **5.24** | 499.5 | 10.7 | 471.1 | 540.2 | 10.7 | 0.0 | 499.6 | 16.9 | 439.9 | 548.7 | 16.9 | 0.0 | 499.5 | 10.6 | 459.4 | 527.4 | 10.6 | 0.0 |
| **6.40** | 499.1 | 13.3 | 459.3 | 537.7 | 13.3 | 0.0 | 500.3 | 23.3 | 439.8 | 569.2 | 23.3 | 0.0 | 501.6 | 22.5 | 431.3 | 566.1 | 22.5 | 0.0 |
| **9.60** | 499.7 | 23.6 | 435.7 | 605.5 | 23.5 | 0.0 | N/A | | | | | 0.0 | 505.9 | 46.1 | 365.6 | 658.8 | 46.4 | 0.0 |
| **p_0_=0.05** |  |  |  |  |  |  |  |  |  |  |  |  |  |  |  |  |  |  |
| **4.71** | 499.2 | 14.1 | 454.3 | 538.3 | 14.1 | 0.0 | 499.9 | 13.8 | 447.1 | 541.0 | 13.8 | 0.0 | 500.6 | 14.0 | 449.0 | 537.9 | 14.0 | 0.0 |
| **5.24** | 499.2 | 17.5 | 443.0 | 549.7 | 17.5 | 0.0 | 499.6 | 16.9 | 439.9 | 548.7 | 16.9 | 0.0 | 499.1 | 16.9 | 444.5 | 555.2 | 16.9 | 0.0 |
| **6.40** | 500.4 | 23.8 | 433.6 | 589.1 | 23.8 | 0.0 | 500.3 | 23.3 | 439.8 | 569.2 | 23.3 | 0.0 | 501.6 | 22.5 | 431.3 | 566.1 | 22.5 | 0.0 |
| **9.60** | 504.1 | 49.7 | 378.4 | 717.4 | 49.9 | 0.0 | N/A | | | | | 0.0 | 505.9 | 46.1 | 365.6 | 658.8 | 46.4 | 0.0 |

Data are averages of 500 simulations.

Clustered trap configurations were not evaluated at 9.60 trap spacing (J=32 traps) as it was equivalent to a regular trap configuration

**S6. For σ = 10 km, summary estimates of $\hat{N}$ in the regular, clustered, and sequential trap configurations when trap spacing increased** from 4.71 to 9.60 km (J=128 to 32 traps) and N=500.

|  | **Regular** | | | | | | **Clustered** | | | | | | **Sequential** | | | | | |
| --- | --- | --- | --- | --- | --- | --- | --- | --- | --- | --- | --- | --- | --- | --- | --- | --- | --- | --- |
| **p_0_=0.20** | **Mean** | **SD** | **Min** | **Max** | **RMSE** | **MNB** | **Mean** | **SD** | **Min** | **Max** | **RMSE** | **MNB** | **Mean** | **SD** | **Min** | **Max** | **RMSE** | **MNB** |
| **4.71** | 499.9 | 0.3 | 498.0 | 500.0 | 0.3 | 0.0 | 500.0 | 0.2 | 499.0 | 500.0 | 0.2 | 0.0 | 499.9 | 0.2 | 499.0 | 500.0 | 0.2 | 0.0 |
| **5.24** | 499.7 | 0.5 | 497.0 | 500.0 | 0.6 | 0.0 | 499.8 | 0.5 | 498.0 | 500.0 | 0.5 | 0.0 | 499.8 | 0.5 | 498.0 | 500.0 | 0.5 | 0.0 |
| **6.40** | 499.4 | 1.0 | 495.3 | 500.5 | 1.1 | 0.0 | 499.4 | 1.0 | 495.3 | 500.6 | 1.2 | 0.0 | 499.5 | 0.9 | 496.2 | 500.5 | 1.0 | 0.0 |
| **9.60** | 499.6 | 3.1 | 485.5 | 507.6 | 3.1 | 0.0 | N/A | | | | | | 499.3 | 2.7 | 492.1 | 506.5 | 2.7 | 0.0 |
| **p_0_=0.10** |  |  |  |  |  |  |  |  |  |  |  |  |  |  |  |  |  |  |
| **4.71** | 499.6 | 1.1 | 495.8 | 501.1 | 1.1 | 0.0 | 499.5 | 1.0 | 495.3 | 500.7 | 1.1 | 0.0 | 499.4 | 1.0 | 496.2 | 500.6 | 1.1 | 0.0 |
| **5.24** | 499.5 | 1.8 | 492.3 | 503.1 | 1.9 | 0.0 | 499.4 | 1.6 | 492.6 | 502.3 | 1.7 | 0.0 | 499.4 | 1.5 | 494.6 | 502.2 | 1.6 | 0.0 |
| **6.40** | 499.5 | 2.7 | 491.2 | 506.2 | 2.8 | 0.0 | 499.3 | 2.7 | 490.7 | 506.1 | 2.8 | 0.0 | 499.5 | 2.7 | 490.8 | 506.3 | 2.8 | 0.0 |
| **9.60** | 499.6 | 6.8 | 478.1 | 518.6 | 6.8 | 0.0 | N/A | | | | | | 499.0 | 6.3 | 478.8 | 515.5 | 6.4 | 0.0 |
| **p_0_=0.05** |  |  |  |  |  |  |  |  |  |  |  |  |  |  |  |  |  |  |
| **4.71** | 499.6 | 3.0 | 491.3 | 507.2 | 3.0 | 0.0 | 499.7 | 2.9 | 490.0 | 505.9 | 2.9 | 0.0 | 499.3 | 3.0 | 482.3 | 506.3 | 3.1 | 0.0 |
| **5.24** | 499.6 | 4.3 | 485.4 | 511.7 | 4.3 | 0.0 | 499.5 | 3.8 | 487.8 | 508.5 | 3.8 | 0.0 | 499.5 | 3.9 | 485.7 | 509.6 | 4.0 | 0.0 |
| **6.40** | 499.5 | 6.4 | 477.9 | 516.2 | 6.4 | 0.0 | 499.6 | 6.2 | 480.7 | 517.5 | 6.2 | 0.0 | 499.3 | 5.8 | 481.9 | 515.2 | 5.9 | 0.0 |
| **9.60** | 500.0 | 12.7 | 461.4 | 531.3 | 12.7 | 0.0 | N/A | | | | | | 498.9 | 12.6 | 461.5 | 532.0 | 12.7 | 0.0 |

Data are averages of 500 simulations.

Clustered trap configurations were not evaluated at 9.60 trap spacing (J=32 traps) as it was equivalent to a regular trap configuration

**S7. For σ = 1 km, summary of estimates of $\hat{\sigma}$ in the regular, clustered and sequential trap configurations when trap spacing increased from 4.71 to 9.60 km (J=128 to 32 traps) and N=500.**

|  | **Regular** | | | | | | **Clustered** | | | | | | **Sequential** | | | | | |
| --- | --- | --- | --- | --- | --- | --- | --- | --- | --- | --- | --- | --- | --- | --- | --- | --- | --- | --- |
| **p_0_=0.20** | **Mean** | **SD** | **Min** | **Max** | **RMSE** | **MNB** | **Mean** | **SD** | **Min** | **Max** | **RMSE** | **MNB** | **Mean** | **SD** | **Min** | **Max** | **RMSE** | **MNB** |
| **4.71** | 1.00 | 0.07 | 0.80 | 1.22 | 0.07 | -0.01 | 1.00 | 0.07 | 0.81 | 1.27 | 0.07 | -0.01 | 1.00 | 0.08 | 0.76 | 1.25 | 0.08 | -0.01 |
| **5.24** | 0.98 | 0.10 | 0.57 | 1.30 | 0.10 | 0.00 | 1.01 | 0.10 | 0.76 | 1.40 | 0.10 | -0.03 | 1.00 | 0.10 | 0.71 | 1.32 | 0.10 | -0.02 |
| **6.40** | 0.98 | 0.17 | 0.49 | 1.39 | 0.17 | -0.03 | 0.99 | 0.14 | 0.64 | 1.60 | 0.14 | -0.02 | 0.99 | 0.14 | 0.57 | 1.38 | 0.14 | -0.02 |
| **9.60** | 0.99 | 0.24 | 0.46 | 1.78 | 0.24 | -0.08 | 0.00 | 0.00 | 0.00 | 0.00 | 0.00 | 0.00 | 1.01 | 0.50 | 0.49 | 10.76 | 0.25 | -0.09 |
| **p_0_=0.10** |  |  |  |  |  |  |  |  |  |  |  |  |  |  |  |  |  |  |
| **4.71** | 1.00 | 0.13 | 0.57 | 1.41 | 0.13 | -0.03 | 1.01 | 0.14 | 0.63 | 1.52 | 0.15 | -0.05 | 0.99 | 0.14 | 0.67 | 1.45 | 0.14 | -0.03 |
| **5.24** | 0.96 | 0.19 | 0.50 | 1.76 | 0.20 | -0.01 | 1.02 | 0.20 | 0.63 | 1.83 | 0.20 | -0.08 | 1.00 | 0.17 | 0.62 | 1.69 | 0.17 | -0.05 |
| **6.40** | 0.98 | 0.24 | 0.50 | 1.51 | 0.24 | -0.07 | 1.00 | 0.27 | 0.52 | 2.02 | 0.27 | -0.11 | 0.96 | 0.24 | 0.53 | 2.63 | 0.24 | -0.05 |
| **9.60** | 1.06 | 0.90 | 0.36 | 16.53 | 0.89 | -0.33 | 0.00 | 0.00 | 0.00 | 0.00 | 0.00 | 0.00 | 1.26 | 2.40 | 0.42 | 38.35 | 2.30 | -0.92 |
| **p_0_=0.05** |  |  |  |  |  |  |  |  |  |  |  |  |  |  |  |  |  |  |
| **4.71** | 0.97 | 0.24 | 0.38 | 1.89 | 0.24 | -0.05 | 1.06 | 0.28 | 0.59 | 1.98 | 0.28 | -0.18 | 1.01 | 0.24 | 0.55 | 2.44 | 0.24 | -0.10 |
| **5.24** | 0.93 | 0.36 | 0.36 | 5.56 | 0.36 | -0.04 | 1.09 | 0.36 | 0.46 | 2.19 | 0.37 | -0.27 | 1.05 | 0.49 | 0.53 | 8.52 | 0.48 | -0.21 |
| **6.40** | 1.02 | 0.32 | 0.39 | 2.90 | 0.31 | -0.13 | 1.11 | 0.64 | 0.47 | 8.25 | 0.63 | -0.38 | 1.20 | 1.84 | 0.45 | 29.97 | 1.60 | -0.62 |
| **9.60** | 1.45 | 2.44 | 0.26 | 19.77 | 2.16 | -1.26 | 0.00 | 0.00 | 0.00 | 0.00 | 0.00 | 0.00 | 4.38 | 32.63 | 0.28 | 529.05 | 24.02 | -8.28 |

Data are averages of 500 simulations.

**S8. For σ = 5 km, summary of estimates of $\hat{\sigma}$ in the regular, clustered and sequential trap configurations when trap spacing increased** from 4.71 to 9.60 km (J=128 to 32 traps) and N=500.

|  | **Regular** | | | | | | **Clustered** | | | | | | **Sequential** | | | | | |
| --- | --- | --- | --- | --- | --- | --- | --- | --- | --- | --- | --- | --- | --- | --- | --- | --- | --- | --- |
| **p_0_=0.20** | **Mean** | **SD** | **Min** | **Max** | **RMSE** | **MNB** | **Mean** | **SD** | **Min** | **Max** | **RMSE** | **MNB** | **Mean** | **SD** | **Min** | **Max** | **RMSE** | **MNB** |
| **4.71** | 5.00 | 0.04 | 4.89 | 5.16 | 0.04 | 0.01 | 5.00 | 0.04 | 4.85 | 5.11 | 0.04 | 0.01 | 5.00 | 0.04 | 4.87 | 5.13 | 0.04 | 0.00 |
| **5.24** | 5.00 | 0.05 | 4.86 | 5.19 | 0.05 | 0.00 | 5.00 | 0.05 | 4.85 | 5.14 | 0.05 | 0.00 | 5.00 | 0.05 | 4.86 | 5.16 | 0.05 | 0.00 |
| **6.40** | 5.00 | 0.06 | 4.84 | 5.19 | 0.06 | 0.00 | 5.00 | 0.06 | 4.83 | 5.17 | 0.06 | 0.00 | 5.01 | 0.06 | 4.82 | 5.21 | 0.06 | -0.01 |
| **9.60** | 5.00 | 0.10 | 4.71 | 5.31 | 0.10 | 0.00 | 0.00 | 0.00 | 0.00 | 0.00 | 0.00 | 0.00 | 5.00 | 0.09 | 4.77 | 5.28 | 0.09 | 0.00 |
| **p_0_=0.10** |  |  |  |  |  |  |  |  |  |  |  |  |  |  |  |  |  |  |
| **4.71** | 5.00 | 0.06 | 4.86 | 5.31 | 0.06 | 0.00 | 5.00 | 0.07 | 4.77 | 5.22 | 0.07 | 0.01 | 5.00 | 0.07 | 4.83 | 5.20 | 0.07 | 0.00 |
| **5.24** | 5.00 | 0.08 | 4.77 | 5.27 | 0.08 | 0.01 | 5.00 | 0.08 | 4.78 | 5.24 | 0.08 | 0.00 | 5.00 | 0.08 | 4.80 | 5.27 | 0.08 | -0.01 |
| **6.40** | 5.00 | 0.10 | 4.73 | 5.27 | 0.10 | 0.01 | 4.99 | 0.10 | 4.74 | 5.33 | 0.10 | 0.02 | 5.01 | 0.10 | 4.72 | 5.33 | 0.10 | -0.02 |
| **9.60** | 5.00 | 0.17 | 4.54 | 5.53 | 0.17 | 0.00 | 0.00 | 0.00 | 0.00 | 0.00 | 0.00 | 0.00 | 4.99 | 0.16 | 4.56 | 5.45 | 0.16 | 0.02 |
| **p_0_=0.05** |  |  |  |  |  |  |  |  |  |  |  |  |  |  |  |  |  |  |
| **4.71** | 5.00 | 0.10 | 4.73 | 5.34 | 0.10 | 0.00 | 4.99 | 0.10 | 4.66 | 5.47 | 0.10 | 0.02 | 5.00 | 0.10 | 4.68 | 5.28 | 0.10 | 0.00 |
| **5.24** | 4.99 | 0.13 | 4.64 | 5.39 | 0.13 | 0.02 | 5.00 | 0.12 | 4.65 | 5.35 | 0.12 | -0.01 | 5.00 | 0.13 | 4.59 | 5.39 | 0.13 | -0.01 |
| **6.40** | 4.99 | 0.17 | 4.49 | 5.48 | 0.17 | 0.01 | 4.99 | 0.17 | 4.55 | 5.49 | 0.17 | 0.03 | 5.01 | 0.17 | 4.51 | 5.51 | 0.17 | -0.02 |
| **9.60** | 4.99 | 0.29 | 3.96 | 5.87 | 0.29 | 0.01 | 0.00 | 0.00 | 0.00 | 0.00 | 0.00 | 0.00 | 4.98 | 0.30 | 4.07 | 5.77 | 0.30 | 0.04 |

Data are averages of 500 simulations.

**S9. For σ = 10 km, summary of estimates of $\hat{\sigma}$ in the regular, clustered and sequential trap configurations when trap spacing increased** from 4.71 to 9.60 km (J=128 to 32 traps) and N=500.

|  | **Regular** | | | | | | **Clustered** | | | | | | **Sequential** | | | | | |
| --- | --- | --- | --- | --- | --- | --- | --- | --- | --- | --- | --- | --- | --- | --- | --- | --- | --- | --- |
| **p_0_=0.20** | **Mean** | **SD** | **Min** | **Max** | **RMSE** | **MNB** | **Mean** | **SD** | **Min** | **Max** | **RMSE** | **MNB** | **Mean** | **SD** | **Min** | **Max** | **RMSE** | **MNB** |
| **4.71** | 9.99 | 0.06 | 9.84 | 10.17 | 0.06 | 0.02 | 10.00 | 0.05 | 9.81 | 10.16 | 0.05 | 0.01 | 10.00 | 0.05 | 9.84 | 10.17 | 0.05 | 0.00 |
| **5.24** | 10.00 | 0.06 | 9.81 | 10.15 | 0.06 | 0.00 | 10.00 | 0.06 | 9.76 | 10.20 | 0.06 | 0.01 | 9.99 | 0.06 | 9.84 | 10.17 | 0.06 | 0.03 |
| **6.40** | 10.00 | 0.07 | 9.76 | 10.21 | 0.07 | 0.01 | 10.00 | 0.08 | 9.78 | 10.25 | 0.08 | -0.01 | 10.00 | 0.07 | 9.77 | 10.23 | 0.07 | 0.01 |
| **9.60** | 10.00 | 0.11 | 9.65 | 10.36 | 0.11 | 0.01 | 0.00 | 0.00 | 0.00 | 0.00 | 0.00 | 0.00 | 10.00 | 0.11 | 9.69 | 10.28 | 0.11 | 0.01 |
| **p_0_=0.10** |  |  |  |  |  |  |  |  |  |  |  |  |  |  |  |  |  |  |
| **4.71** | 9.99 | 0.08 | 9.74 | 10.24 | 0.08 | 0.02 | 10.00 | 0.08 | 9.74 | 10.21 | 0.08 | 0.00 | 10.00 | 0.07 | 9.80 | 10.26 | 0.07 | -0.01 |
| **5.24** | 10.00 | 0.09 | 9.74 | 10.23 | 0.09 | -0.01 | 10.00 | 0.09 | 9.67 | 10.28 | 0.09 | 0.00 | 9.99 | 0.09 | 9.76 | 10.28 | 0.09 | 0.02 |
| **6.40** | 9.99 | 0.11 | 9.64 | 10.38 | 0.11 | 0.02 | 10.00 | 0.11 | 9.67 | 10.35 | 0.11 | 0.00 | 9.99 | 0.11 | 9.70 | 10.37 | 0.11 | 0.02 |
| **9.60** | 10.00 | 0.17 | 9.32 | 10.56 | 0.17 | 0.00 | 0.00 | 0.00 | 0.00 | 0.00 | 0.00 | 0.00 | 10.00 | 0.17 | 9.58 | 10.59 | 0.17 | -0.01 |
| **p_0_=0.05** |  |  |  |  |  |  |  |  |  |  |  |  |  |  |  |  |  |  |
| **4.71** | 10.00 | 0.11 | 9.68 | 10.40 | 0.11 | 0.01 | 10.00 | 0.11 | 9.58 | 10.33 | 0.11 | 0.01 | 10.00 | 0.11 | 9.67 | 10.44 | 0.11 | -0.01 |
| **5.24** | 10.01 | 0.13 | 9.59 | 10.34 | 0.13 | -0.03 | 10.00 | 0.13 | 9.54 | 10.45 | 0.13 | 0.01 | 9.99 | 0.14 | 9.57 | 10.44 | 0.14 | 0.03 |
| **6.40** | 9.99 | 0.16 | 9.53 | 10.46 | 0.16 | 0.02 | 10.00 | 0.18 | 9.48 | 10.48 | 0.18 | 0.01 | 10.00 | 0.15 | 9.57 | 10.53 | 0.15 | 0.00 |
| **9.60** | 9.99 | 0.26 | 9.31 | 10.71 | 0.26 | 0.03 | 0.00 | 0.00 | 0.00 | 0.00 | 0.00 | 0.00 | 10.01 | 0.26 | 9.34 | 10.75 | 0.26 | -0.05 |

Data are averages of 500 simulations.
