# Supplementary material for: Correction: Trap Configuration and Spacing Influences Parameter Estimates in Spatial Capture-Recapture Models
Source: PLoS One. 2015 Oct 23;10(10):e0141634. doi: 10.1371/journal.pone.0141634 (PMC4619801; doi:10.1371/journal.pone.0141634)
Supplement: S1 Appendix — (DOCX) [file pone.0141634.s002.docx]

**Appendix 1**

Varying trap spacing without constraining the area within which traps are placed should produce RMSE curves that are quadratic, achieving a minimum at intermediate values of trap spacing (1.5- 2.5 σ) indicating an optimal trap spacing (e.g., [43] and Fig 10.1 in [14]). However, this pattern does not hold when the area within which traps are placed is constrained. For example, in Table 7 (reproduced below) we see a monotone increase in RMSE with increasing effective trap spacing at p_0_=0.20 and 0.10. This is because effective trap spacing is a combination of varying both σ and J (the number of traps) within the fixed area. RMSE did not monotonically increase when p_0_=0.05 and σ=1 km because an increasing number of iterations were discarded. For a given value of σ, we achieved an increase in effective trap spacing by decreasing the number of traps, producing a concomitant decrease in expected sample size and hence increase in RMSE. In doing so, simulation conditions (i.e., a fixed area) limited the flexibility in designing trap configurations for assessing trap spacing.

Table 7. RMSE values of estimators of $\hat{\boldsymbol{N}}$ as effective trap spacing increased from 0.47σ to 10.67σ under the regular trap configuration and across all baseline detection probabilities (p0=0.20, 0.10, and 0.05). RMSE monotonically increased when p_0_=0.20 and 0.10, due to simultaneous decreases in σ from 10 km to 1 km and decreases in the number of traps, J, from 128 to 32. RMSE (italicized) did not monotonically increase when p_0_=0.05 and σ = 1 km because 4, 12, 53 and 172 iterations were discarded from J=128, 94, 64, and 32, respectively.

|  | |  | p_0_ | | |
| --- | --- | --- | --- | --- | --- |
| σ (km) | J | Effective Trap Spacing (σ) | 0.20 | 0.10 | 0.05 |
| 10 | 128 | 0.47 | 0.3 | 1.1 | 3.0 |
| 10 | 96 | 0.52 | 0.6 | 1.9 | 4.3 |
| 10 | 64 | 0.64 | 1.1 | 2.8 | 6.4 |
| 10 | 32 | 1.07 | 2.2 | 5.7 | 12.2 |
| 5 | 128 | 0.94 | 6.4 | 9.4 | 14.1 |
| 5 | 96 | 1.05 | 7.3 | 10.7 | 17.5 |
| 5 | 64 | 1.28 | 8.8 | 13.3 | 23.8 |
| 5 | 32 | 2.13 | 12.6 | 24.2 | 49.9 |
| 1 | 128 | 4.71 | 75.6 | 183.8 | *507.1* |
| 1 | 96 | 5.24 | 167.2 | 353.7 | *845.8* |
| 1 | 64 | 6.40 | 285.4 | 571.5 | *788.6* |
| 1 | 32 | 10.67 | 495.9 | 998.4 | *647.1* |

**Appendix 2**

The objective of the simulation study was to assess regular, clustered, and sequential trap configurations for a study area of fixed size, motivated by interest in estimating abundance or density for a particular area. It is natural to think of a regular design to have equal trap spacing in the X- and Y- directions. However, with a fixed and square study area, this is generally only possible when the number of traps is a perfect square. Thus, our 8 x 8 (J=64) design achieved constant X- and Y- spacing of 6.40 km. When the number of traps was not a perfect square, we maintained trap spacing in the X direction at 6.40 km but varied the trap spacing in the Y direction from 3.01 km to 14.94 km. This was necessary in order to place 16, 12, and 4 traps in each of the eight trap columns to obtain J=128, 96, and 32, respectively (Figure 4 in paper). While regularly-spaced trap configurations may be preferable for simulations, it is usually not possible in actual field work to achieve such designs due to constraints resulting from a fixed study area and limitations such as access to private land. Therefore, some alternative rule to constructing regular trap configurations is needed.

As an alternative to requiring constant X- and Y-spacing, we can seek ‘regular’ designs which achieve a uniform coverage of the study area in some quantitative sense. Thus, we will define uniform spatial coverage with respect to a criterion q(D) for design D. One possibility is to define a numerical objective function that describes spatial coverage (e.g., space-filling designs, see [Royle et al. 2014], sec. 10.5.5). A heuristic criterion to define uniform spatial coverage is the average probability of capture for any individual in a prescribed area (either the study area of interest, or the state-space used to define the point process of activity centers), $\bar{p}$. Thus, good designs should maximize $\bar{p}$. $\bar{p}$ is conditional on trap locations, so it can be used to evaluate and compare trap configurations.

We compared the $\bar{p}$ of the regular trap configurations used in our simulations to $\bar{p}$ of other intuitive trap configurations with square or nearly-square trap outlines of the study area when J=128, 96, and 32. To generate the alternative configurations, we started with a square design with constant X- and Y- spacing of traps and thinned those square designs in a systematic manner. We thinned the square grids to achieve designs of size J=128, 96, and 32 by removing either 1) exterior corner traps, 2) interior corner traps, or 3) interior traps (Figures 1, 2, and 3, respectively). We did not consider $\bar{p}$ when J=64 because traps were equidistantly spaced, and calculated $\bar{p}$ with σ=5 and p_0_=0.20. The trap configurations used in our simulations, with different X- and Y- spacing, resulted in $\bar{p}$ values that were comparable to those of the square and nearly-square designs (Table 1). When J=128 and 96, differences between configurations were no greater than 3.3% (Figures 4 and 5), and in fact configurations used in the simulations had larger values of $\bar{p}$ than when either corner or interior corners were removed. When J=32, differences in $\bar{p}$ were less than 1% (Figure 6). Although the regular trap configurations used in the simulations do not produce the maximum possible $\bar{p}$ over the larger state-space of the study because of the fixed study area size, this comparison of trap configurations using the $\bar{p}$ heuristic indicates that they are comparable to that of alternative configurations when the size of the study area is fixed.


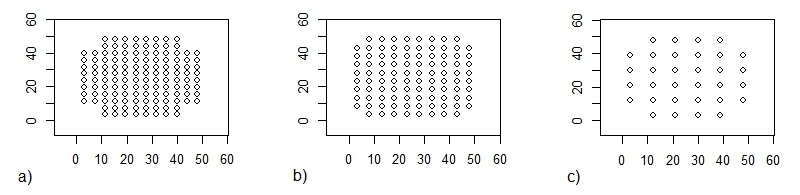


Figure 1. Trap configurations of J=128 (a), 96 (b), and 32 (c), in which corner traps were removed. Traps are equidistant in both the X and Y directions. When J=128, 4 traps from each corner of a 12x12 grid were removed; when J=96, one trap from each corner of a 10x10 grid were removed; when J=32, one trap from each corner of a 6x6 grid were removed.

.


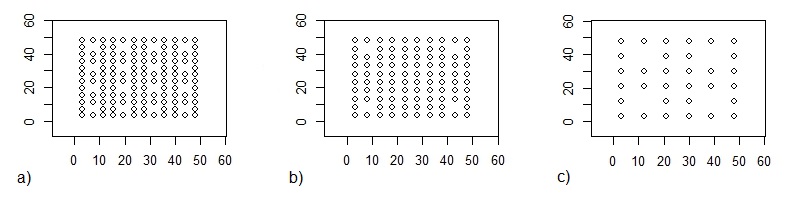


Figure 2. Trap configurations of J=128 (a), 96 (b), and 32 (c), in which interior corner traps were removed from each quarter of the configurations. Red rectangles show an example quarter in each configuration. When J=128, 4 interior corner traps were removed from each quarter; when J=96 and 32, one interior corner was removed from each quarter.


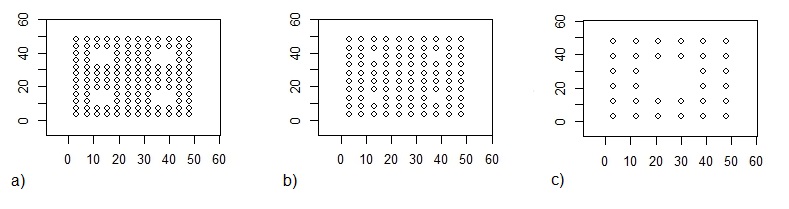


Figure 3. Trap configurations of J=128 (a), 96 (b), and 32 (c), in which interior center traps were removed. Red rectangles show example quarters in the J=129 and 96 configurations. When J=128, the 4 center traps of each quarter were removed; when J=96, the center trap of each quarter was removed. When J=32, the 4 center traps of the trapping grid were removed, as removing the center trap from each quarter would have been equivalent to removing the interior corner trap (see Figure 2c).

Table 1. Comparison of $\bar{\boldsymbol{p}}$ values of the four regular configurations with J=128, 96, and 32 traps. “Simulation” refers to the trap configuration used in the simulations; “Corners” refers to the trap configuration with traps removed from the exterior corners; “Interior Corners” refers to the trap configuration with interior traps removed; “Interior Centers” refers to the trap configuration with interior traps at either the centers of the grid quarters (J=128, 96) or trapping grid (J=32) removed.

|  | $\bar{\boldsymbol{p}}$ | | | |
| --- | --- | --- | --- | --- |
| J | Simulation | Corners | Interior Corners | Interior Centers |
| 128 | 0.5390 | 0.5064 | 0.5357 | 0.5405 |
| 96 | 0.4614 | 0.4531 | 0.4594 | 0.4624 |
| 32 | 0.2078 | 0.2056 | 0.2086 | 0.2079 |


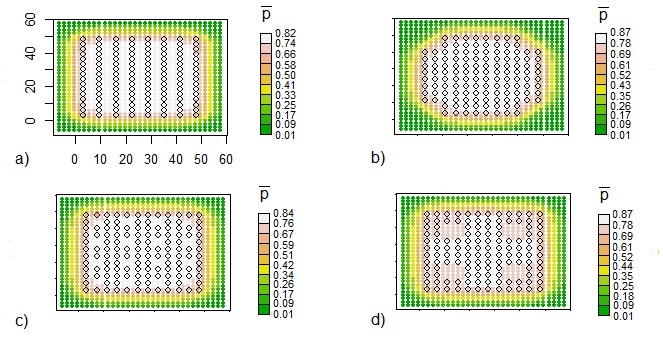


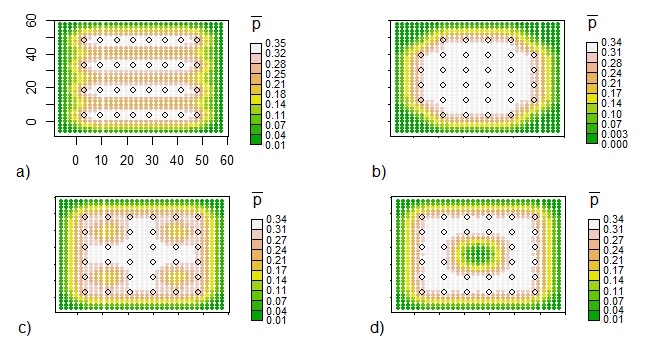

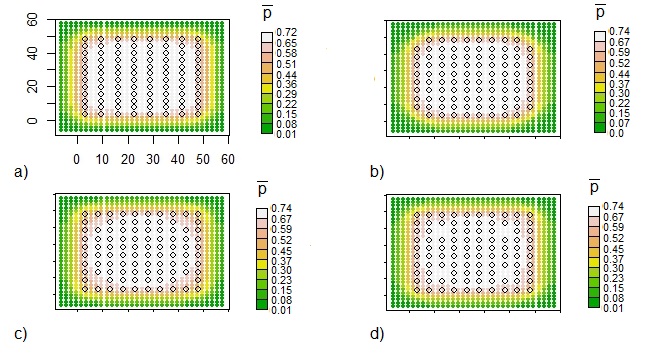


Figure 5. Spatial plots of $\bar{\boldsymbol{p}}$ across the state-space when J=96 traps in the original (a) and three alternative regular configurations, where either corner (b), interior corner (c), or interior center (d) traps were removed. $\bar{\boldsymbol{p}}$ ranged from 0.01 (green) to 0.72-0.74 (white). Open circles show the trap configurations.

Figure 4. Spatial plots of $\bar{\boldsymbol{p}}$ across the state-space when J=128 traps in the original (a) and three alternative regular configurations, where either corner (b), interior corner (c), or interior center (d) traps were removed. $\bar{\boldsymbol{p}}$ ranged from 0.01 (green) to 0.82-0.87 (white). Open circles show the trap configurations.

Figure 6. Spatial plots of $\bar{\boldsymbol{p}}$ across the state-space when J=32 traps in the original (a) and three alternative regular configurations, where either corner (b), interior corner (c), or interior center (d) traps were removed. $\bar{\boldsymbol{p}}$ ranged from 0.000 (green) to 0.34-0.35 (white). Open circles show the trap configurations.


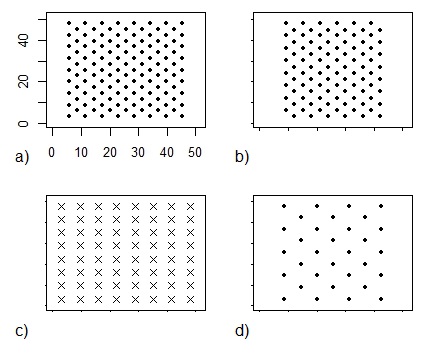
In separate simulations, we created regular configurations with constant X- and Y- spacing for J=128, 96, and 32 by allowing the study area size to vary in width. To achieve this, we offset half the traps in each configuration in the Y-direction (Figure 7). Equidistant trap spacings were thus 3.96, 4.23, 6.40, and 7.92 km when J=128, 96, 64, and 32, respectively. This led to a trap geometry that differed from when J=64, which having a square number of traps, had a square grid with no offset traps. This resulted in an interesting pattern in the precision of $\hat{N}$. While we expected precision to decrease with increasing trap spacing, resulting from fewer traps, the precision of $\hat{N}$ actually increased when the number of traps decreased from 128 to 64 traps in five of six combinations of σ and p_0_ (Table 2). In other words, RMSE of $\hat{N}$ decreased when J=64 when we expected it to increase. This can be attributed to an increase in the number of detected individuals when J=64, even though the mean number of captures per individual decreased as expected (Table 3). For example, at p0=0.20 and σ=5km, an average of 436 individuals were detected when J=64, which is 65 more individuals detected than when J=96 and 12 more individuals than even when J=128. These results provide further evidence that estimation of population parameters can be sensitive to sampling design, and that geometry of trap configurations merits further research.

Figure 7. Trap configurations of J=128 (a), 96 (b), 64 (c), and 32 (d) in which traps were equidistantly spaced 3.96, 4.23, 6.40, and 7.92 km, respectively. This was achieved with J=128, 96, and 32 traps (i.e., when J was not a perfect square number) by allowing the X extent of the study area to vary and by offsetting half of the traps in the Y- direction.

Table 2. $\hat{\boldsymbol{N}}$ when traps were equidistantly spaced at 3.96,4.23,6.40,or 7.92 km across a varying study area size by decreasing the number of traps from J=128 to J=32, when σ = 5 km and 10 km, and p0=0.20,0.10, and 0.05. Except when σ=5 and p0=0.05, root mean squared error (RMSE) was smaller than when trap spacing was closer. These cases are highlighted in red.

|  |  | σ = 5 km | | | σ = 10 km | | |
| --- | --- | --- | --- | --- | --- | --- | --- |
|  |  | Mean | SD | RMSE | Mean | SD | RMSE |
| J | p_0_=0.20 |  |  |  |  |  |  |
| 128 | 3.96 | 499.55 | 9.58 | 9.58 | 499.55 | 0.69 | 0.82 |
| 96 | 4.23 | 498.56 | 13.40 | 13.47 | 499.49 | 1.75 | 1.82 |
| 64 | 6.40 | 499.00 | 8.80 | 8.80 | 499.40 | 1.00 | 1.10 |
| 32 | 7.92 | 499.57 | 16.63 | 16.62 | 499.64 | 4.24 | 4.25 |
|  | p_0_=0.10 |  |  |  |  |  |  |
| 128 | 3.96 | 500.01 | 12.85 | 12.83 | 499.51 | 1.78 | 1.85 |
| 96 | 4.23 | 498.62 | 16.90 | 16.94 | 499.63 | 3.56 | 3.58 |
| 64 | 6.40 | 499.10 | 13.30 | 13.30 | 499.50 | 2.70 | 2.80 |
| 32 | 7.92 | 499.06 | 24.69 | 24.68 | 499.75 | 8.63 | 8.62 |
|  | p_0_=0.05 |  |  |  |  |  |  |
| 128 | 3.96 | 499.84 | 17.07 | 17.06 | 499.68 | 3.95 | 3.96 |
| 96 | 4.23 | 499.58 | 21.88 | 21.86 | 499.37 | 6.86 | 6.89 |
| 64 | 6.40 | 500.40 | 23.80 | 23.80 | 499.50 | 6.40 | 6.40 |
| 32 | 7.92 | 500.22 | 44.53 | 44.48 | 499.43 | 14.80 | 14.79 |

Table 3. Mean number of detected individuals (Inds) and captures per individual (Captures) when the number of equidistantly-spaced traps decreased from J=128 to 96, 64, and 32 traps, when σ = 5 km and 10 km. While the number of captures per individual decreased with fewer traps (and increasing trap spacing), the number of detected individuals increased when the number of traps decreased from J=96 to 64. The cases highlighted in red correspond to the cases in Table 2 where RMSE decreased despite fewer numbers of traps (and increased trap spacing).

|  |  | σ =5 km | | σ =10 km | |
| --- | --- | --- | --- | --- | --- |
|  |  | Inds | Captures | Inds | Captures |
| J | p_0_=0.20 |  |  |  |  |
| 128 | 3.96 | 423.5 | 10.9 | 499.6 | 34.6 |
| 96 | 4.23 | 370.9 | 9.3 | 497 | 26.3 |
| 64 | 6.40 | 435.9 | 5.3 | 499.1 | 16.7 |
| 32 | 7.92 | 335 | 3.4 | 484.2 | 8.9 |
|  | p_0_=0.10 |  |  |  |  |
| 128 | 3.96 | 385.3 | 5.9 | 496.5 | 17.3 |
| 96 | 4.23 | 332.3 | 5.1 | 487.3 | 13.3 |
| 64 | 6.40 | 382.9 | 3.0 | 492.7 | 8.4 |
| 32 | 7.92 | 268.4 | 2.1 | 450.1 | 4.7 |
|  | p_0_=0.05 |  |  |  |  |
| 128 | 3.96 | 336.2 | 3.4 | 484.3 | 8.8 |
| 96 | 4.23 | 284.4 | 3.0 | 461.9 | 7.0 |
| 64 | 6.40 | 299.4 | 1.9 | 466.8 | 4.4 |
| 32 | 7.92 | 186.6 | 1.5 | 385.2 | 2.8 |
